# A unique hormonal recognition feature of the human glucagon-like peptide-2 receptor

**DOI:** 10.1038/s41422-020-00442-0

**Published:** 2020-11-25

**Authors:** Wen Sun, Li-Nan Chen, Qingtong Zhou, Li-Hua Zhao, Dehua Yang, Huibing Zhang, Zhaotong Cong, Dan-Dan Shen, Fenghui Zhao, Fulai Zhou, Xiaoqing Cai, Yan Chen, Yan Zhou, Sarina Gadgaard, Wijnand J. C. van der Velden, Suwen Zhao, Yi Jiang, Mette M. Rosenkilde, H. Eric Xu, Yan Zhang, Ming-Wei Wang

**Affiliations:** 1 The CAS Key Laboratory of Receptor Research, Shanghai Institute of Materia Medica, Chinese Academy of Sciences, Shanghai, 201203 China; 2 The National Center for Drug Screening, Shanghai Institute of Materia Medica, Chinese Academy of Sciences, Shanghai, 201203 China; 3 University of Chinese Academy of Sciences, Beijing, 100049 China; 4 Department of Biophysics and Department of Pathology of Sir Run Run Shaw Hospital, Zhejiang University School of Medicine, Hangzhou, Zhejiang 310058 China; 5 iHuman Institute, ShanghaiTech University, Shanghai, 201210 China; 6 School of Basic Medical Sciences, Fudan University, Shanghai, 200032 China; 7 School of Pharmacy, Fudan University, Shanghai, 201203 China; 8 Department of Biomedical Sciences, University of Copenhagen, Copenhagen, N, DK-2200 Denmark; 9 School of Life Science and Technology, ShanghaiTech University, Shanghai, 201210 China; 10 MOE Frontier Science Center for Brain Research and Brain–Machine Integration, Zhejiang University School of Medicine, Hangzhou, Zhejiang 310058 China; 11 Key Laboratory of Immunity and Inflammatory Diseases of Zhejiang Province, Hangzhou, Zhejiang 310058 China; 12 Zhejiang Laboratory for Systems and Precision Medicine, Zhejiang University Medical Center, Hangzhou, Zhejiang 311121 China

**Keywords:** Cryoelectron microscopy, Hormone receptors

## Abstract

Glucagon-like peptides (GLP-1 and GLP-2) are two proglucagon-derived intestinal hormones that mediate distinct physiological functions through two related receptors (GLP-1R and GLP-2R) which are important drug targets for metabolic disorders and Crohn’s disease, respectively. Despite great progress in GLP-1R structure determination, our understanding on the differences of peptide binding and signal transduction between these two receptors remains elusive. Here we report the electron microscopy structure of the human GLP-2R in complex with GLP-2 and a G_s_ heterotrimer. To accommodate GLP-2 rather than GLP-1, GLP-2R fine-tunes the conformations of the extracellular parts of transmembrane helices (TMs) 1, 5, 7 and extracellular loop 1 (ECL1). In contrast to GLP-1, the N-terminal histidine of GLP-2 penetrates into the receptor core with a unique orientation. The middle region of GLP-2 engages with TM1 and TM7 more extensively than with ECL2, and the GLP-2 C-terminus closely attaches to ECL1, which is the most protruded among 9 class B G protein-coupled receptors (GPCRs). Functional studies revealed that the above three segments of GLP-2 are essential for GLP-2 recognition and receptor activation, especially the middle region. These results provide new insights into the molecular basis of ligand specificity in class B GPCRs and may facilitate the development of more specific therapeutics.

## Introduction

GLP-2R belongs to class B GPCR subfamily, which is mainly expressed in the gut, pancreas and brain.^1^ Its endogenous ligand is GLP-2 (GLP-2(1–33)), a member of the glucagon-like peptide family that also includes GLP-1 and glucagon. GLP-2 is encoded by the proglucagon gene and becomes active after post-translational processing by prohormone convertases. As a gastrointestinal hormone, it mainly regulates intestinal epithelial cell growth and functions that are crucial for digestion and absorption of nutrients.^2^ Similar to GLP-1, GLP-2 is rapidly inactivated by dipeptidyl peptidase 4 in vivo.^3^ Clinically, GLP-2 analogue, teduglutide, is used to treat short bowel syndrome and Crohn’s disease. Other therapeutic indications, including colitis, pediatric gastrointestinal disorders and inflammation of the intestinal mucosa, are currently under development.^4^ Recent studies in animal models have shown that GLP-2 also exerts beneficial effects on glucose homeostasis,^5,6^ spontaneous hypertension^7–9^ and depression.^10–12^ This prompts a renewed interest in examining the role of this peptide beyond the gastrointestinal tract.

The cryo-electron microscopy (cryo-EM) emerges as a primary methodology to determine the structure of GPCR–G protein complexes starting with the GLP-1R^13^ and the calcitonin receptor,^14^ followed by eight other class B GPCRs in complex with various G proteins.^15–20^ These structures suggest that class B GPCRs have a common activation pattern which basically coincides with the previously proposed two-domain binding model.^21^ Although class B receptors have roughly the same activation mode, the molecular details are receptor specific, especially in the ligand-binding region. Therefore, exploring the recognition mechanism between GLP-2 and GLP-2R is of interest for further understanding the mechanism of ligand recognition and receptor activation among class B GPCRs. In this study, we employed cryo-EM to determine the high-resolution structure of the human GLP-2R in complex with G_s_ protein. Based on our prior experience,^22^ NanoBiT strategy^23,24^ was used in this study to stabilize the GLP-2–GLP-2R–G_s_ complex and strengthen the interactions between GLP-2R and Gβ, resulting in a cryo-EM structure at a global resolution of 3.0 Å (Supplementary information, Table S1). Our data provide a rational template to facilitate the design of better GLP-2 analogues for therapeutic use and expand our knowledge on the biology of this receptor family.

## Results and discussion

### Structure determination

We used NanoBiT strategy^22–24^ to stabilize the complex, in which GLP-2R was directly attached to LgBiT and rat Gβ was linked to HiBiT with a 15-amino acid linker in between. Transiently transfected HEK 293T cells showed that the C-terminus-truncated GLP-2R(1–490)-LgBiT had a 6-fold stronger interaction with Gβ-SmBiT than the wild-type (WT) receptor(1–553)-LgBiT (Supplementary information, Fig. S1a, b). When SmBiT was replaced by HiBiT, the luminescence signal detected between GLP-2R and Gβ was 10-fold stronger (Supplementary information, Fig. S1c). A haemagglutinin (HA) signal peptide was added to the N-terminus of GLP-2R to increase the expression level. In addition to linking with LgBiT, a TEV protease site and an OMBP (optimized maltose binding protein)-MBP tag were attached to the C-terminus (Supplementary information, Fig. S2a). The OMBP tag is codon-optimized on the basis of MBP to increase its expression in insect cells. At the same time, using OMBP-MBP tag can enhance affinity between the protein complex and amylose resin in order to obtain more complexes. Rat Gβ1 was attached to HiBiT with a 15-amino acid linker between them (Supplementary information, Fig. S2b). To form an active G protein-coupled complex, HA-GLP-2R(1–490)-LgBiT-TEV-OMBP-MBP was co-expressed with a dominant-negative bovine Gα_s_ (DNGα_s_), rat Gβ1-HiBiT and bovine Gγ2 in *Sf*9 insect cells. Compared with the protein expressed without NanoBiT strategy, the yield decreased but the stability increased (Supplementary information, Fig. S1d, e). Negative-staining EM visualization displayed more particles with similar size (Supplementary information, Fig. S1f).

A large-scale purification was subsequently performed using this approach. Cells were stimulated by 20 μM GLP-2 together with the camelid-derived nanobody Nb35 to stabilize the interface between Gα_s_ and Gβ.^25^ The complex was solubilized in lauryl maltose neopentyl glycol (LMNG) and cholesteryl hemi succinate (CHS), treated with TEV enzyme to remove tags from GLP-2R and then isolated by amylose resin. It was further purified by size-exclusion chromatography for cryo-EM specimen preparation (Supplementary information, Fig. S3a, b). The production of cAMP and ligand-binding ability of our modified hGLP-2R construct were reduced presumably caused by its lower expression level (~73% of WT; Supplementary information, Fig. S3c, d and Table S2).

Frozen-hydrated GLP-2–GLP-2R–G_s_ complexes were imaged using a Titan Krios microscope (Supplementary information, Fig. S4a). 2D classification showed clear secondary structure features and random distribution of particles in different directions enabling high-resolution cryo-EM map reconstruction (Supplementary information, Fig. S4b). A total of 284,669 particles were selected after consecutive 3D classifications, which yielded a cryo-EM density map with a global resolution of 3.0 Å (Supplementary information, Fig. S4c, d).

### Overall architecture

**Fig. 1:**
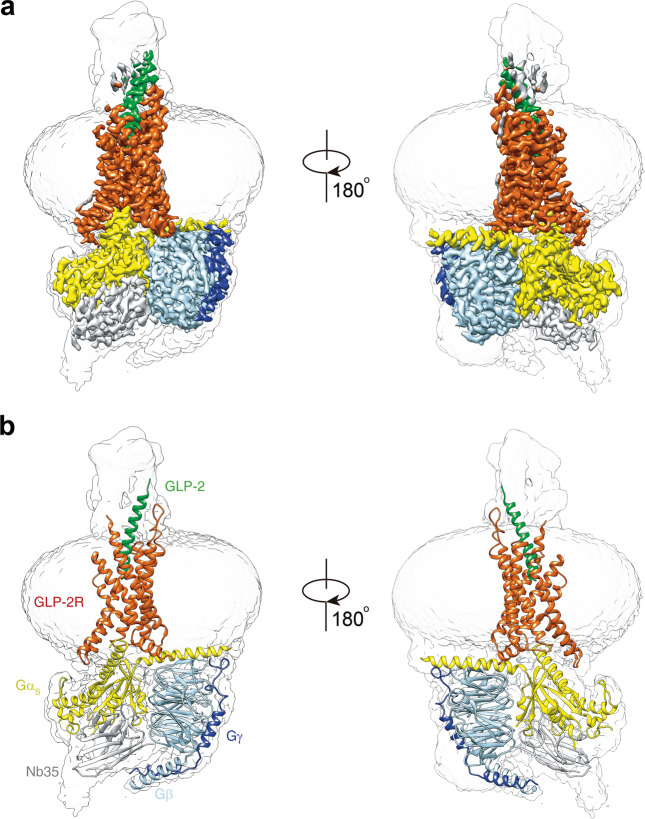
The overall cryo-EM structure of GLP-2–GLP-2R–G_s_ complex. **a** Cut-through view of cryo-EM density map illustrating the GLP-2–GLP-2R–G_s_ complex and the transparent disc-shaped micelle. Receptor density is shown in orange red, GLP-2 in forest green, Gα_s_ in yellow, Gβ in light blue, Gγ in medium blue and Nb35 in light gray. **b** Model of the complex as a cartoon, with GLP-2 as helix in forest green. The receptor is shown in orange red, Gα_s_ in yellow, Gβ in light blue, Gγ in medium blue and Nb35 in light gray.

In the cryo-EM structure of the GLP-2–GLP-2R–G_s_ complex, most of the receptor core, G protein subunits and the first 31 amino acids of GLP-2 were observed with high-resolution features, thereby allowing for accurate modeling of side-chain rotamers (Fig. 1; Supplementary information, Fig. S5). The receptor extracellular domain (ECD) was omitted from the final reported structure because of the relatively low resolution owning to its intrinsic flexibility, which is a general feature in most reported class B GPCR–G_s_ complex structures.

The overall structure of active GLP-2R–G_s_ complex is similar to other class B GPCR–G_s_ complexes such as GLP-1–GLP-1R–G_s_,^13^ ExP5–GLP-1R–G_s_,^17^ glucagon–GCGR–G_s_,^18^ LA-PTH–PTH1R–G_s_^19^ and GHRH–GHRHR–G_s_,^26^ with root mean square deviation (RMSD) values of 1.76, 1.05, 1.55, 1.09 and 0.95 Å for the whole complex, respectively. When pointing toward the glucagon receptor subfamily, notable conformational differences were observed in the extracellular half of GLP-2R where the peptide hormone binds, especially in TM1, ECL1 and TM7, indicating that GLP-2R utilizes a distinct peptide-recognition mode specific for GLP-2, but not for GLP-1, glucagon or GHRH.

### Molecular recognition

**Fig. 2:**
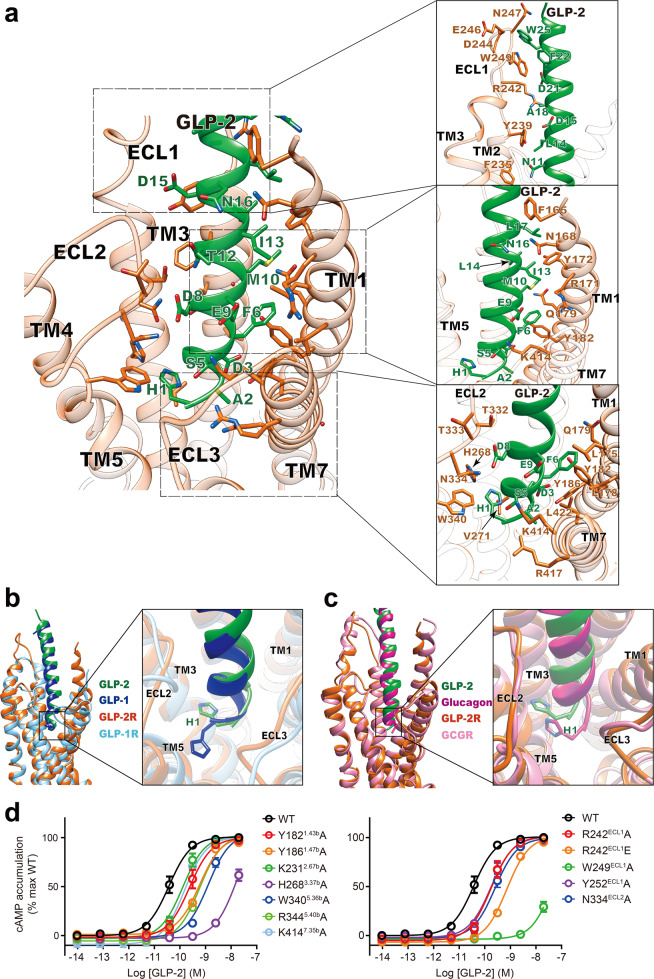
Molecular recognition of GLP-2 by GLP-2R. **a** Extensive contacts between GLP-2R (sticks with orange red carbons) and GLP-2 (sticks with forest green carbons), showing that the N-terminal residue His^1P^ can interact with H268^3.37b^, V271^3.40b^ and W340^5.36b^, together with other interactions between agonist and receptor (left). The interactions focus on the ECL1 of GLP-2R and GLP-2 (right). **b**, **c** Structural comparison of the TMD binding pockets among GLP-2–GLP-2R–G_s_, GLP-1–GLP-1R–G_s_,^13^ and glucagon–GCGR–G_s_.^19^ GLP-2 in forest green, GLP-2R in orange red; GLP-1 in blue, GLP-1R in light blue; glucagon in magenta and GCGR in light magenta. Receptor ECD and G protein are omitted for clarity. **d** Effects of mutation in the peptide-binding pocket on cAMP accumulation. cAMP levels were measured in WT receptor and alanine-mutated TMs and ECLs. Data shown are means ± SEM of at least three independent experiments.

**Fig. 3:**
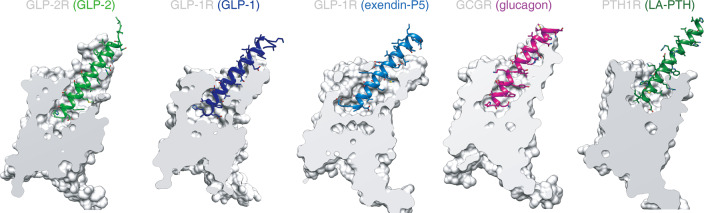
Comparison of the peptide-binding pocket of GLP-2R with other class B GPCRs. The binding cavity of GLP-2R is compared with that of GLP-1-bound GLP-1R (PDB code: 5VAI),^13^ ExP5-bound GLP-1R (PDB code: 6B3J),^17^ glucagon-bound GCGR (PDB code: 6LMK)^18^ and LA-PTH-bound PTH1R (PDB code: 6NBF).^19^

The activated GLP-2R structure shows that GLP-2 is stably anchored in its position through an extensive network of interactions that involves TMs 1, 2, 3, 5 and 7, and ECLs 1, 2 and 3 (Fig. 2). The N-terminus of GLP-2, His^1P^ (P indicates that the residue belongs to GLP-2), which is located above the conserved central polar network of class B GPCRs, is oriented toward TM3 and forms a hydrogen bond with H268^3.37b^ (Wootten numbering in superscript^27^) and hydrophobic contacts with V271^3.40b^, W340^5.36b^ and R344^5.40b^ (Fig. 2a). Consistently, the truncated metabolite GLP-2(3–33) acts as a partial agonist with great reduction in binding affinity and potency.^28,29^ Meanwhile, mutants H268A, W340A and R344A decreased GLP-2 potency by 457-, 34- and 16-fold, respectively (Fig. 2d; Supplementary information, Table S3). Despite an identical N-terminal histidine in GLP-2, GLP-1 and glucagon, the side-chain orientations of histidine in the cognate receptors are different: the GLP-2 His^1P^ is more distant from TM5 than that of GLP-1 and closer to TM3 than that of glucagon (Figs. 2, 3). Such difference may arise from the subtle changes in pocket residues at 3.37b (H268 for GLP-2R, Q234 for GLP-1R and Q232 for GCGR), 3.40b (V271 for GLP-2R, V237 for GLP-1R and I235 for GCGR) and ECL2 (N334 for GLP-2R, R299 for GLP-1R and N298 for GCGR). Another key residue in the N-terminus of GLP-2 is Asp^3P^, whose side-chain forms a salt bridge with K231^2.67b^ and a hydrogen bond with Y186^1.47b^. Alanine substitutions in Y186^1.47b^ and K231^2.67b^ had a moderate effect (4–16 fold) on GLP-2 potency (Fig. 2d).

The middle region of GLP-2 forms both a polar network with several charged and polar amino acids in TM7 (K414^7.35b^ and R417^7.38b^) and ECL2 (T332^ECL2^ and N334^ECL2^) via Gly^4P^, Ser^5P^, Asp^8P^, Glu^9P^ and Asn^11P^ and a complementary nonpolar network with TM1 (R171^1.32b^, Y172^1.33b^, L175^1.36b^, L178^1.39b^ and Q179^1.40b^) via Phe^6P^, Met^10P^, Ile^13P^ and Leu^14P^ (Fig. 2a). Intriguingly, we observed one water molecule bridging Ser^7P^ and D232^2.68b^, which is absent in the GLP-1–GLP-1R structure.^13^ When comparing the active GLP-2R structure with the GLP-1-bound GLP-1R structure,^13^ the extracellular end of TM1 in GLP-2R is extended by six additional residues, thereby bending toward GLP-2 with the formation of additional hydrogen bond and hydrophobic contacts. These interactions further stabilize the peptide conformation in the bound state and may contribute to specific recognition of GLP-2.

Partial unwinding of the short α-helix elevates ECL1 to an extended conformation that stands upwards in line with TM2 and TM3, thereby engaging the C-terminal half of GLP-2 through an extensive interface spanning from Leu^14P^ to Trp^25P^ (Figs. 2a, 3). Specifically, Asp^21P^ forms a salt bridge with R242^ECL1^, while Trp^25P^ forms a hydrogen bond with D244^ECL1^ and pi-pi stacking with W249^ECL1^, which was stabilized by Phe^22P^. Besides, Leu^14P^ and Ala^18P^ pack against Y239^ECL1^, R242^ECL1^ and Y252^ECL1^. These interactions strengthen the binding and function of GLP-2, consistent with the decreased potencies of R242A (by 5-fold), R242E (by 20-fold), W249A (by 1552-fold) and Y252A (by 5-fold) (Fig. 2d). Similar effects of mutations were observed with the binding of teduglutide (Table 1). Together, these results highlight the importance of ECL1 in GLP-2 recognition and receptor activation.

**Table 1:** Binding of GLP-2 and teduglutide with GLP-2R mutants^a^.

	Human GLP-2 (1–33)		Teduglutide	
Receptor mutant	pIC_50_	% Binding of WT^b^	pIC_50_	% Binding of WT
WT (1–553)	8.5 ± 0.07	99.2 ± 2.3	8.8 ± 0.05	99.7 ± 1.9
Y182^1.43^A	9.1 ± 1.2	3.4 ± 1.0***	9.2 ± 0.8	3.2 ± 1.3***
Y186^1.47^A	N.B.^c^	N.B.	N.B.	N.B.
K231^2.67^A	N.B.	N.B.	N.B.	N.B.
R242^ECL1^E	N.B.	N.B.	N.B.	N.B.
R242^ECL1^A	8.0 ± 0.5	4.2 ± 0.6***	8.7 ± 0.8	5.1 ± 1.5***
D244^ECL1^A	8.6 ± 0.2	69.1 ± 4.9***	9.0 ± 0.2	77.5 ± 5.7***
N247^ECL1^A	8.2 ± 0.1	130.7 ± 4.5***	8.6 ± 0.1	135.2 ± 4.4***
W249^ECL1^A	9.0 ± 1.2	2.2 ± 1.2***	8.8 ± 1.7	2.9 ± 2.0***
Y252^ECL1^A	8.4 ± 0.8	8.3 ± 1.8***	9.0 ± 0.6	6.3 ± 1.7***
H268^3.37^A	8.0 ± 1.4	2.1 ± 0.8***	9.7 ± 3.2	0.7 ± 1.8***
N334^ECL2^ A	9.5 ± 1.8	2.4 ± 1.3***	9.7 ± 2.7	2.0 ± 2.1***
W340^5.36^A	7.1 ± 0.4	7.3 ± 0.6***	8.8 ± 0.7	8.6 ± 1.8***
R344^5.40^A	8.6 ± 0.5	5.0 ± 0.8***	9.3 ± 0.6	3.5 ± 0.8***
K414^7.35^ A	8.5 ± 0.2	15.2 ± 1.0***	8.7 ± 0.2	13.9 ± 0.8***
ECL1 (GLP-1R)^d^	8.9 ± 1.7	3.4 ± 1.3***	7.8 ± 0.7	1.2 ± 0.5***
ECL1 (poly-alanine)^d^	8.2 ± 1.3	3.0 ± 1.1***	N.B.	N.B.

***P  < 0.001.

^a^All data were fitted with a three-parameter logistic curve to obtain pIC_50_ values. Data represent means ± SEM of at least three independent experiments performed in duplicate. One-way ANOVA and Dunnett’s post hoc tests were used to determine statistical differences.

^b^*WT* wild-type.

^c^*N.B.* no binding of the radioligand.

^d^GLP-2R mutants with ECL1 (residues 236–257) substituted by the corresponding segment of GLP-1R or poly-alanine are labeled as ECL1 (GLP-1R) and ECL1 (poly-alanine), respectively.

### G protein coupling

In our model of the GLP-2R–G_s_ complex, G_s_ protein is anchored by the α5 helix of Gα_s_ (GαH5), thereby fitting to the cytoplasmic cavity of the transmembrane domain (TMD) involving TMs 2, 3, 5, 6, and 7 (Supplementary information, Fig. S6). Structural comparison of the GLP-2R–G_s_ complex with that of other class B GPCRs reveals substantial similarity in the G protein-binding interface, consistent with a common mechanism of G_s_ protein engagement. However, many receptor-specific interactions were observed. Y391^GαH5^ is an essential residue in determining G protein coupling specificity by forming H-bond in class A GPCRs,^30^ however, this interaction was not observed among available class B GPCR–G protein complexes. Most strikingly, Y391^GαH5^ formed a tight hydrogen bond with a water molecule, which connects to E281^3.50b^ and H214^2.50b^ in the GLP-2R–G_s_ complex (Supplementary information, Fig. S6), a phenomenon that was not observed among other class B GPCRs. Polar interactions also occurred between K368^5.64b^ and Q384^GαH5^, H372^ICL3^ and R385^GαH5^ as well as R382^6.37b^ and E392^GαH5^. The ICL2 of GLP-2R formed additional hydrophobic interactions with GαH5. There were also limited interactions between GαH5 subunit and helix 8 of the receptor (E392^GαH5^, N440^8.47b^ and G441^8.48b^). These receptor-specific interactions may contribute to the G protein specificity of GLP-2R.

### Ligand specificity

**Fig. 4:**
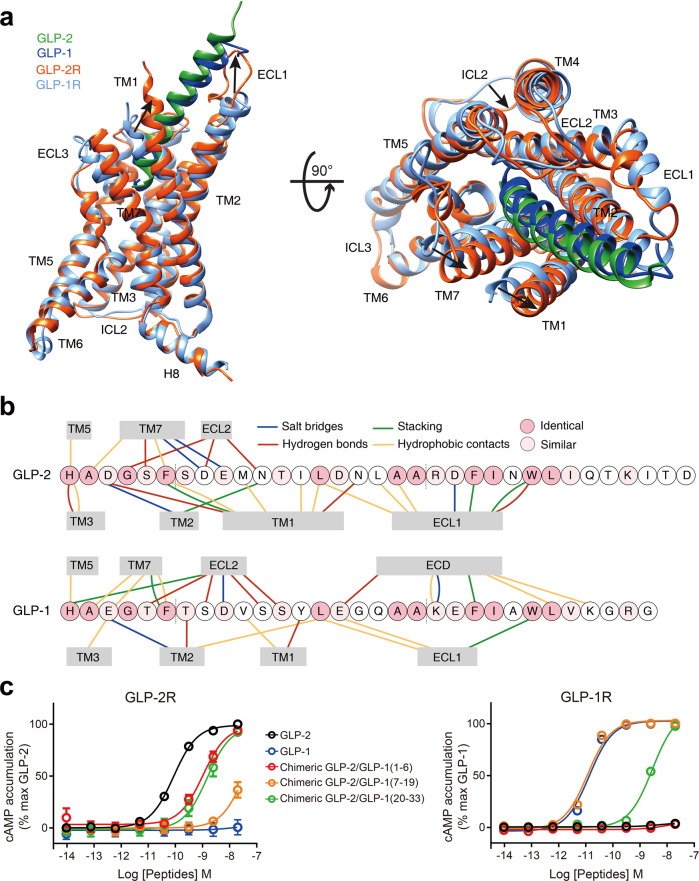
Basis of ligand specificity between GLP-2R and GLP-1R. **a** Structural comparison of GLP-2–GLP-2R–G_s_ and GLP-1–GLP-1R–G_s_^.13^ Receptor ECD and G protein are omitted for clarity. **b** Schematic diagram of interactions between peptide and receptor. Conserved residues in GLP-2 and GLP-1 are highlighted in pink, while those similar are shown in light pink. Length of gray shading box illustrates the interface area between peptide and corresponding segment. **c** Signaling profiles of the chimeric GLP-2/GLP-1 peptides. Replacement of the N-terminus (residues 1–6), middle region (residues 7–19) and C-terminus (residues 20–33) of GLP-2 by corresponding segment of GLP-1 was named as chimeric GLP-2/GLP-1(1–6), GLP-2/GLP-1(7–19) and GLP-2/GLP-1(20–33), respectively. Dose-response curves of peptide-induced cAMP accumulation for GLP-2R were generated and graphed as means ± SEM from three independent experiments.

GLP-2 and GLP-1 share high sequence similarity (69.7%) and have almost identical residues in two regions: the N-terminus (first six residues in GLP-2) that penetrates into the TMD core, and the C-terminus (residues 18–33) that is recognized by the ECD and ECL1 of the receptor. On the contrary, the central region of the peptide mainly interacts with TMs 1, 2, 7, ECL1 and ECL2 of the receptor and differs to that of GLP-1 (Fig. 4; Supplementary information, Fig. S7a, b).

Structural comparison of GLP-2–GLP-2R–G_s_ and GLP-1–GLP-1R–G_s_^13^ complexes reveals distinct features of their ligand-binding pockets (Figs. 3, 4; Supplementary information, Fig. S7c–e). Superimposing the TMD of GLP-2R with that of GLP-1R shows that the intracellular portions of both receptors, except ICL2 and ICL3, overlap very well, whereas the extracellular parts of TM1, TM5 and TM7 as well as all ECLs have different conformations and side-chain orientations (Fig. 4a; Supplementary information, Fig. S7c–e). Consistently, the N-terminal part of GLP-2 is reoriented relative to GLP-1. Furthermore, the rest of GLP-2 is elevated vertically (~0.5 Å) and moves toward TM1–TM2 by 1–3 Å, allowing the extensive interaction with ECL1. Overall, the interface area measured by buried solvent-accessible surface of GLP-2–GLP-2R TMD is 3050 Å^2^, significantly larger than that of GLP-1–GLP-1R TMD (2297 Å^2^).

The orientation of the N-terminal histidine between GLP-1 and GLP-2 is rather different (Fig. 2b; Supplementary information, Fig. S7c). GLP-1 faces toward TM5 to form cation-pi stacking and hydrogen bond with the rotated down side-chain of R299^ECL2^, while that of W306^5.36b^ and R310^5.40b^ switch out from the receptor core to accommodate the approaching of histidine. However, by replacing arginine with threonine, the ECL2 of GLP-2R is no longer able to dip into the receptor core and becomes distant from the N-terminal histidine of GLP-2. Therefore, W340^5.36b^ and R344^5.40b^ of GLP-2R rotate inwards. Together with the inward movement of the extracellular part of TM5, they push the N-terminus of GLP-2 away from TM5 to reside closely with TM3, where H268^3.37b^ contributes a hydrogen bond to stabilize the conformation (Supplementary information, Fig. S7c). Compared to GLP-1, the middle region of GLP-2 has more extensive contacts with the ligand-binding pocket (Fig. 4b). The interface areas between TMD and GLP-2 contributed by TM1 and TM7 are 864 Å^2^ and 432 Å^2^, respectively, markedly larger than those between TMD and GLP-1 (330 Å^2^ and 303 Å^2^, respectively). Specifically, in the extracellular half of GLP-2R, TM1 forms at least one additional helical turn, thereby moving toward TM2 (5 Å, measured by Cα of the residue at 1.33b) and bending down toward GLP-2. This ultimately leads to a much larger interaction pattern for GLP-2 (ranging from Asp^3P^ to Asn^16P^) than for GLP-1, where only two residues of GLP-1 interact with TM1.

Meanwhile, side-chain orientations of TM1 residues are reorganized (Supplementary information, Fig. S7d). For example, Y182^1.43b^ is reoriented ~90° from an outside facing position to a position pointing to TM2 and engages T-shaped stacking with Phe^6P^ of GLP-2. Accompanying TM1 movement to TM2, the extracellular tip of TM7 moves toward TM1 and the side-chain of K414^7.35b^ points to GLP-2 with formation of a salt bridge with Glu^9P^. In the C-terminus of GLP-2, we observed that it forms a more extensive interaction with the ECL1 of GLP-2R compared to the GLP-1–GLP-1R ECL1 interaction (interface area = 791 Å^2^ vs 424 Å^2^). Different from a 2.5-turn α-helix of ECL1 in both activated GLP-1R and GCGR, the ECL1 of GLP-2R partially unwinds vertically in line with TM2 and TM3, providing additional interactions not seen in its counterparts (Figs. 3, 4a; Supplementary information, Fig. S7e). Alternatively, the α1-helix of GLP-1R ECD is in close contact with the C-terminus of GLP-1. Because the ECD structure of GLP-2R is unavailable, its impact on the structure and dynamics remains elusive.

Considering the uneven sequence conservation and distinct binding modes of GLP-2 and GLP-1, we designed chimeric GLP-2/GLP-1 peptides to explore the molecular basis of ligand specificity. As shown in Fig. 4c, replacement of the first six residues of GLP-2 by those of GLP-1 (i.e., double mutant D^3P^E and S^5P^T) decreased cAMP accumulation by 12-fold compared to the native peptide, suggesting that subtle substitution may directly alter receptor activation. In addition, chimeric GLP-2 whose C-terminal fragment (residues 20–33) was replaced with that of GLP-1 displayed a 17-fold potency reduction in GLP-2R signaling but successfully elicited GLP-1R-mediated cAMP accumulation (Fig. 4c). Replacement of the GLP-2 middle region (residues 7–19) that mainly interacts with TM1, TM7 and ECL1 by the corresponding part of GLP-1, almost abolished GLP-2R-mediated cAMP accumulation but fully rescued that induced by GLP-1R (Fig. 4c), indicative of a fundamental role of this region in determining the specificity for ligand recognition.

### Conformational changes

**Fig. 5:**
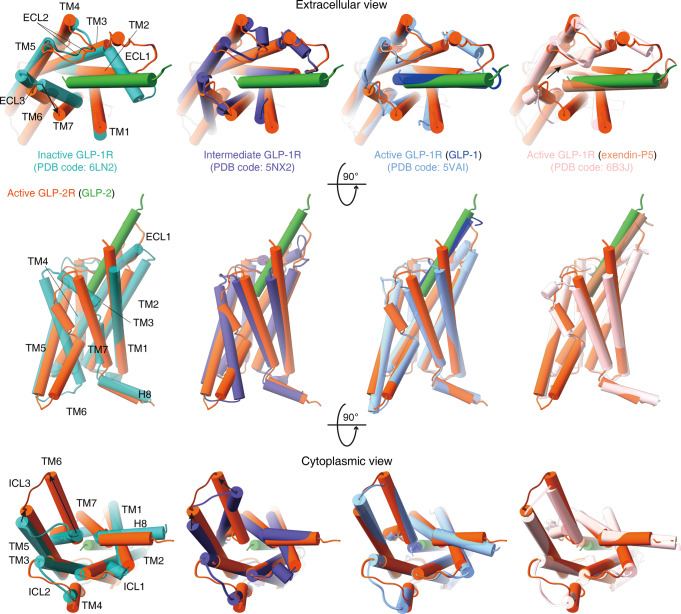
Conformational changes upon GLP-2R activation. Comparison of active GLP-2R with inactive, agonist-bound and both agonist-bound and G protein-coupled active GLP-1R.^13,17,32–35^ Receptor ECD and G protein are omitted for clarity.

Triggered by GLP-2 binding and G protein coupling, GLP-2R would presumably undergo significant conformational changes. It shares a high degree of sequence similarity in the TMD region with GLP-1R (61%) that has been studied extensively both structurally and functionally.^31^ Thus, inactive, intermediate and active GLP-1R structures published previously^13,17,32–35^ provide good references for the present study. Superimposing the TMD of GLP-2R with that of GLP-1R reveals that activated GLP-2R shows a similar conformation to that of GLP-1-bound or ExP5-bound GLP-1R–G_s_ complexes^13,17^ (Cα RMSD = 1.41 and 0.91 Å, respectively), distinct from inactive GLP-1R^33^ (Cα RMSD = 1.79 Å). Further comparison demonstrates that diversified peptide–receptor interactions converge to induce conserved and extensive conformational transition of the TMD of GLP-2R and GLP-1R (Fig. 5).

In the extracellular half of GLP-2R, accompanying the unwinding of the last two turns of TM6, TM7 moves outward and bends toward TM6 via a conserved pivot point G429^7.50b^, which expands the TMD-binding pocket to accommodate peptide entrance and connection. Meanwhile, TM1 moves inwards and folds down toward GLP-2, which contributes both polar and hydrophobic contacts to stabilize the binding. Furthermore, different from the ordered α-helical ECL1 in inactive GLP-1R^33^ and β-hairpin in inactive GCGR^36^ that cover the binding pocket and probably block peptide insertion, ECL1 of active GLP-2R, GLP-1R and GCGR simultaneously move outward and stand upwards in line with TM2 and TM3. Together with inward movement of α-helical extended TM2, raised ECL1 of GLP-2R forms extensive interaction with the C-terminal half of GLP-2 (Fig. 4a, b) and was shown to be essential for peptide binding and receptor activation (Fig. 4c). As far as the intracellular half is concerned, the positions of TM1–2–3 of active GLP-2R resemble that of active GLP-1R (Fig. 5). The most profound conformational change is the sharp kink in the middle of TM6, leading to an outward movement of the intracellular portion of TM6 as measured by Cα carbon of Lys^6.35b^ (19.0 Å, similar to that of other G_s_-coupled class B receptors). In contrast, in the absence of G protein coupling, the binding of peptide 5 promotes a rigidly outward shift of the intracellular tip of TM6 of GLP-1R by 12.1 Å without a sharp kink,^34^ thereby creating an intracellular crevice for G protein coupling. For PTH1R^37^ and GCGR^38^ (whose crystal structures were determined with PGS domain fusion in ICL3 and T4L fusion at ICL2, respectively), agonist binding failed to promote the outward movement of the intracellular half of TM6 as seen in the inactive state, probably due to the fusion. With more structural and dynamics studies on class B GPCRs, the activation process from inactive to active states via an intermediate state, i.e., allosteric communication between extracellular peptide binding and intracellular G protein coupling, will be better understood.

### Role of ECL1

**Fig. 6:**
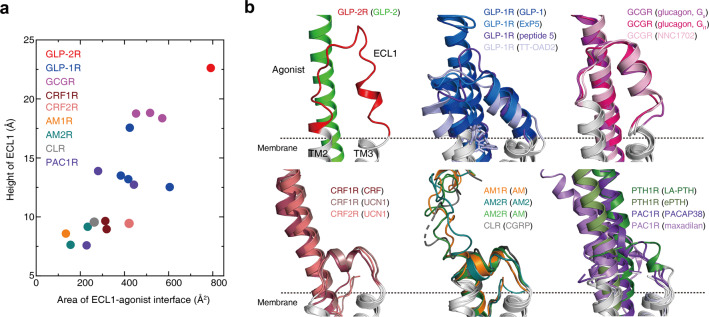
Family-wide comparison of ECL1 conformations. **a** Scatter plot of ECL1 from 9 class B GPCRs. *x* axis shows the interface area between ECL1 and agonist, and *y* axis is the height of ECL1. **b** Structural comparison of diversified ECL1 conformations among class B GPCRs. All structures are superimposed on the GLP-1-bound GLP-1R (PDB: 5VAI)^13^ using the Cα carbons of the residues in the TM2–3–4. The locations of the membrane planes predicted by OPM (Orientations of Proteins in Membranes)^54^ are shown as black dashed lines. The height of ECL1 is defined as the maximum vertical distance of Cα carbon relative to the membrane layer, while the interface areas were calculated using freeSASA.^53^

Relative to ECL2 and ECL3, ECL1 in class B GPCRs is the most divergent extracellular loop with flexible lengths, ranging from 8 (CRF2R) to 27 (PTH1R) residues. Inspired by the intriguing ECL1 conformation of GLP-2R, we performed structural comparison of ECL1 across class B GPCRs to unveil its functionality (Fig. 6). Among 20 agonist-bound structures from 9 receptors, we calculated the height of ECL1 (defined as the maximum vertical distance of Cα carbon relative to the membrane layer) and the ECL1–agonist interface area. We found that GLP-2R has the most protruded ECL1 with a height of 22.6 Å and this ECL1 closely attaches to GLP-2 with the largest interface area of 791 Å^2^. The ECL1 of all activated GLP-1R and GCGR structures consistently adopt α-helical conformation bridging extended TM2 and TM3 helices by two short linkers to form extensive interactions with bound agonists. Remarkably, significant conformational changes of ECL1 upon receptor activation were observed relative to their inactive conformations: (i) in the inactive GCGR, ECL1 forms a β-hairpin conformation and further compacts β-sheet with the stalk region; and (ii) in the inactive GLP-1R, ECL1 moves toward TM1 and connects ECD in its closed TMD-interacting conformation. As far as CRF1R, CRF2R, AM1R, AM2R and CLR are concerned, their ECL1 are relatively short (consisting of 8–11 residues) and can only form one-turn α-helix to connect the adjacent transmembrane helices TM2 and TM3, contributing limited contacts with the peptides. PTH1R has the longest ECL1, but it is unstructured in both LA-PTH–PTH1R–G_s_^19^ and ePTH–PTH1R^37^ structures, probably owing to the great mobility of PTH1R’s ECD and specific binding mode, consistent with the mutagenesis studies.^37,39,40^ To date, four PAC1R structures were reported, namely, PACAP38–PAC1R–G_s_ (3.0 Å),^20^ PACAP–PAC1R–mini-G_s_ (3.9 Å),^41^ PACAP38–PAC1R–G_s_ (3.5 Å)^42^ and maxadilan–PAC1R–G_s_ (3.6 Å).^42^ The ECL1 was not resolved in the first two structures, and the third map showed that the ECL1 closely interacted with PACAP38 (pituitary adenylate cyclase activating polypeptide-38) without detailed residue contact information due to limited resolution.^42^ Interestingly, maxadilan, a peptidic PAC1R agonist, reorganized the ECD orientation and reshaped the ligand-binding pocket conformation, including ECL1^42^ (Fig. 6b). Collectively, these data demonstrate the diversity and flexibility of ECL1 among class B GPCRs.

To further explore the functional importance of ECL1 in ligand binding and receptor activation, we performed mutagenesis studies on this part of the receptor (Table 1; Supplementary information, Fig. S8 and Table S4). Replacement of GLP-2R ECL1 by poly-alanine abolished the ability of GLP-2 to elicit cAMP response, while replacement by that of GLP-1R diminished its potency by 196-fold. Meanwhile, single-point mutations on deeply buried residues (R242, W249 and Y252) in the interface between GLP-2 and ECL1 also demonstrated negative impact on ligand binding (Supplementary information, Table S5).

In conclusion, the structure of the GLP-2–GLP-2R–G_s_ complex delineates detailed interactions between GLP-2 and GLP-2R that account for the peptide binding specificity, and provides new insights into the structural reorganization of class B GPCRs upon activation. Combined with previous research on the GLP-1R, our results reveal that subtle differences in the sequence of TMD binding pocket and the consequent diversified conformational changes of the extracellular half accommodate peptide binding and empower the receptor to be specifically trigged by the cognate hormone. This complex structure points to an essential role of ECL1 in peptide recognition and receptor activation, in addition to that of ECD. This work also highlights the applicability of the NanoBiT tethering strategy to stabilize GPCR–G protein complexes for structural studies.

## Materials and methods

### Constructs

WT human GLP-2R was modified to include a HA signal peptide at the N-terminus. LgBiT and an OMBP-MBP tag were added to the C-terminus of the receptor with a TEV site between them. A bovine Gα_s_ was used with G226A and A366S mutations to stabilize the complex;^43^ rat Gβ1 was attached to HiBiT with a 15-amino acid linker between them; bovine Gγ2 was also used in cloning. All of the above constructs were generated in pFastBac and pcDNA3.1 vectors for protein expression in insect cells and functional assays in mammalian cells, respectively.

### Protein expression

The complex was co-expressed in *Sf*9 cells (Expression System, Davis, USA) grown in ESF-921 serum-free medium (Expression System) at 27 °C, 120 rpm. Baculovirus was added at a proportion of 1:1:1:1 for HA-GLP-2R(1–490)-LgBiT-TEV-OMBP-MBP, Gα_s_, Gβ-HiBit and Gγ when the cell density reached 3 × 10^6^/mL. After 48 h incubation, the pellet was harvested by PBS using centrifugation at 2000 rpm for 20 min.

### Complex purification

*Sf*9 cells were suspended in 20 mM HEPES, pH 7.4, 100 mM NaCl and 10% (v/v) glycerol in the presence of Protease Inhibitor Cocktail (Topscience, Shanghai, China). Complex was formed by adding 10 mM CaCl_2_, 10 mM MgCl_2_, 1 mM MnCl_2_, 50 mU/mL apyrase (Sigma-Aldrich, Darmstadt, Germany), 20 μM GLP-2, 100 μM TCEP (Sigma-Aldrich) and 10 μg/mL Nb35 into the cell lysate and incubated at room temperature (RT) for 1.5 h. Cell membranes were solubilized by 0.5% (w/v) LMNG (Anatrace, Maumee, USA) supplemented with 0.1% (w/v) CHS  (Anatrace) with 5 μM GLP-2 and Protease Inhibitor Cocktail (Topscience) at 4 °C for 2 h, followed by centrifugation at 65,000× *g* for 30 min at 4 °C. The supernatant was taken to bind with amylose resin (NEB, Ipswich, USA) for 2 h at 4 °C. After packing, the column was washed with buffer containing 20 mM HEPES, pH 7.4, 100 mM NaCl, 10% (v/v) glycerol, 5 μM GLP-2, 25 μM TCEP, 5 mM MgCl_2_, 1 mM MnCl_2_, 0.1% (w/v) LMNG and 0.02% (w/v) CHS first (10 column volumes), and then with decreased concentrations of detergents 0.03% (w/v) LMNG, 0.01% (w/v) GDN and 0.008% (w/v) CHS (20 column volumes). TEV enzyme was added to the resin and kept at 4 °C overnight to remove OMBP-MBP tag. The complex was eluted from the resin and concentrated to 500 μL using a 100 kDa MWCO Amicon Ultra Centrifugal Filter (Merck Millipore, Darmstadt, Germany). Size-exclusion chromatography was carried out by loading the protein sample to Superdex 200 Increase 10/30GL (GE Healthcare, Boston, USA) to obtain the monomer complex. The column was pre-equilibrated with 20 mM HEPES, pH 7.4, 100 mM NaCl, 5 μM GLP-2, 100 μM TCEP, 2 mM MgCl_2_, 0.00075% (w/v) LMNG, 0.00025% (w/v) GDN and 0.0002% (w/v) CHS.

### Nb35 production

*E. coli* BL21 cells were transformed with the plasmid containing Nb35 target gene and were grown in TB medium with 100 μg/mL ampicillin, 1 M MgCl_2_, 2% (w/v) glucose at 37 °C, 180 rpm for 3 h. IPTG (1 M) was added to induce the expression when OD600 reached 0.7–1.2. After 8 h expression, the cell pellet was collected and stored at −80 °C until use. Nb35 was purified by nickel affinity chromatography as previously described,^25^ followed by size-exclusion chromatography using a HiLoad 16/600 Superdex 75 column (GE Healthcare). The column was pre-equilibrated with 20 mM HEPES, pH 7.4 and 100 mM NaCl. Glycerin 30% (v/v) was added to collect Nb35 which was frozen in liquid nitrogen and stored at −80 °C.

### Negative-staining electron microscopy

Purified GLP-2R–G_s_ complex was diluted to 0.012 mg/mL with 20 mM HEPES, pH 7.4, 100 mM NaCl, 5 μM GLP-2, 100 μM TCEP, 2 mM MgCl_2_, 0.00075% (w/v) LMNG, 0.00025% (w/v) GDN and 0.0002% (w/v) CHS. Eight microliters of protein complex and 8 μL uranium formate were applied to a glow-discharged 300-mesh cooper grid (EMCN, Beijing, China) for 1 min and then sucked with filter paper. After grilling the grid with a lamp, the samples were imaged at RT with a Talos L120C electron microscope (FEI, Hillsboro, USA) operated at 120 kV. Images were recorded at magnification of 57,000× and a defocus value of −1 μm.

### Cryo-EM

Three microliters of the purified GLP-2–GLP-2R–G_s_ complex at about 20 mg/mL was applied onto a glow-discharged holey carbon grid (Quantifoil R1.2/1.3), blotted, and subsequently sample-coated grids were vitrified by plunging into liquid ethane using a Vitrobot Mark IV (ThermoFisher Scientific, Waltham, USA). Automatic data collection was performed on a Titan Krios equipped with a Gatan K2 Summit direct electron detector in the Center of Cryo-Electron Microscopy, Zhejiang University (Hangzhou, China). The microscope was operated at 300 kV accelerating voltage, at a nominal magnification of 29,000× in counting mode, corresponding to a pixel size of 1.014 Å. A total of 2296 movies were obtained at a dose rate of about 8 electrons per Å^2^ per second with a defocus ranging from −0.5 to −2.5 μm. The total exposure time was 8 s and intermediate frames were recorded in 0.2 s intervals, resulting in an accumulated dose of 64 electrons per Å^2^ and a total of 40 frames per micrograph.

### Data processing and 3D reconstructions

Dose-fractionated image stacks were subjected to beam-induced motion correction using MotionCor2.1.^44^ A sum of all frames, filtered according to the exposure dose, in each image stack was used for further processing. Contrast transfer function parameters for each micrograph were determined by Gctf v1.06.^45^ Particle selection, 2D and 3D classifications were performed on a binned dataset with a pixel size of 2.028 Å using RELION-3.0-beta2.^46^ Auto-picking yielded 1,498,893 particle projections were subjected to reference-free 2D classification to discard false positive particles or particles categorized in poorly defined classes, producing 485,548 particle projections for further processing. This subset of particle projections was subjected to a round of maximum-likelihood-based three dimensional classifications with a pixel size of 2.028 Å, resulting in one well-defined subsets with 162,932 projections. Further 3D classifications with mask on the complex produced two good subsets accounting for 332,946 particles, which were subsequently subjected to a round of 3D classifications with mask on the receptor. A selected subset containing 284,669 projections was then subjected to 3D refinement and Bayesian polishing with a pixel size of 1.014 Å. After last round of refinement, the final map has an indicated global resolution of 3.0 Å at a Fourier shell correlation (FSC) of 0.143. Local resolution was determined using the Bsoft package with half maps as input maps.^47^

### Model building

The initial template of the TMDs in GLP-2R was generated using SWISS-MODEL.^48^ The models of G_s_ protein and Nb35 from PTH1R complex structure (PDB: 6NBF)^19^ were used as the relevant components of the GLP-2R–G_s_ complex models. While the whole TMDs, G_s_ protein, and Nb35 showed decent density, the ECDs of GLP-2R complex structures displayed very poor density in the whole density maps and were thus omitted in the final structural models. All the models were docked into the EM density map using UCSF Chimera.^49^ Model adjustment and rebuilding were performed in COOT.^50^ Real space and Rosetta refinements were carried out using Phenix software package.^51^ All residues were checked for fitting in electron density, and Ramachandran and rotamer restraints. The refined models exhibited good model-density fitting and model geometry (see Supplementary information, Table S1 for data and model refinement statistics).

### cAMP accumulation

HEK 293T cells were cultured in Dulbecco’s Modified Eagle’s Medium (DMEM) (Gibco, Waltham, USA) supplemented with 10% (v/v) fetal bovine serum (FBS) (Gibco) and 1% (v/v) sodium pyruvate (Gibco) at 37 °C, 5% CO_2_ in a humidified incubator. They were seeded onto 6-well cell culture plates and transiently transfected with different GLP-2R constructs using Lipofectamine 2000 transfection reagent (Invitrogen, Carlsbad, CA, USA). After 24 h, the transfected cells were seeded onto 384-well microtiter plates (3000 cells per well) in HBSS supplemented with 5 mM HEPES, 0.1% (w/v) bovine serum albumin (BSA) and 0.5 mM 3-isobutyl-1- methylxanthine, and stimulated with increasing concentrations of GLP-2 for 30 min at RT. Eu and Ulight were diluted by cAMP detection buffer and added to the plates separately to terminate the reaction. Plates were incubated at RT for 60 min before measuring the fluorescence intensity at 620 nm and 650 nm by an EnVision multilabel plate reader (PerkinElmer, Boston, USA).

### NanoBiT assay

HEK 293T cells were seeded onto 6-well cell culture plates in DMEM containing 10% FBS and 1% sodium pyruvate at 37 °C, 5% CO_2_ before transfection. They were transiently transfected with truncated GLP-2 receptors attached to LgBiT and Gβ-SmBiT (or Gβ-HiBiT) in a ratio of 1:1 using Lipofectamine 2000 transfection reagent (Invitrogen) and incubated for 24 h. After seeding onto 96-well microtiter plates (48,000 cells per well), the medium was replaced by HBSS followed by 30 min incubation at 37 °C. Coelenterazine H (5 μM) was then added to the plates and luminescence signals measured 1 h thereafter using an EnVision multilabel plate reader (PerkinElmer) at 18 s intervals before and after ligand addition (25 °C).

### Oxidative iodination

The GLP-2 radioligand was created by oxidative iodination using ChloramineT to incorporate the iodine isotope [^125^I] at position Tyr10 of human GLP-2(1–33, M10Y). A stepwise oxidation reaction was performed by addition of 6 aliquots of 5 µL ChloramineT (30 µL/mL) with a 1-min interval during constant stirring. The reaction was terminated by the addition of 400 µL phase A (0.1% trifluoracetic acid) before being fractionated into 1 mL volume by reverse-phase high-performance liquid chromatography (RP-HPLC, Waters 600 SimiPrep system) on a C18 column. The iodinated protein was assembled by increasing concentrations of phase B (Acetonitrile + 0.1% TFA) and tested for competitive binding ability.

### Receptor binding

One day before transfection, the COS-7 cells were seeded in 75 cm^2^ culture flasks (3 million cells/flask) in DMEM, containing 3.9 g/L NaHCO_3_ and supplemented with 10% FBS, 1% l-glutamine, 180 units/mL penicillin and 45 µg/mL streptomycin. After an overnight culture at 37 °C, 10% CO_2_ and 95% air humidity, the cells were transiently transfected with different GLP-2R constructs or pcDNA3.1 vector as previously described.^52^ Following 24-h incubation, the cells were transferred to Costa® microtiter plates (150,000 cells/well, PerkinElmer) to achieve a 5%–10% specific binding. Forty-eight hours after transfection, the cells were washed twice in binding buffer (50 mM HEPES, pH 7.4, supplemented with 1 mM CaCl_2_, 5 mM MgCl_2_ and 0.5% (w/v) BSA) and incubated for 15 min at 4 °C. An increasing concentration of unlabeled GLP-2(1–33) or teduglutide (ranging from 0.1 nM to 1 μM) followed by a fixed concentration of [^125^I]GLP-2(1–33, M10Y) (10–15 pM) was added to the cells and incubated for 4 h at 4 °C. The cells were then washed twice in binding buffer (4 °C), lysed and counted for radioactivity using a Wallac Gamma Counter (PerkinElmer).

### Structural analysis

The interface area was calculated by FreeSASA^53^ using the Sharke-Rupley algorithm with a probe radius of 1.2 Å. Structural figures were prepared using UCSF Chimera^49^ and PyMOL (The PyMOL Molecular Graphics System v.2.1). RMSD analysis was performed in PyMOL.

## Supplementary information

Supplementary information fig S1

Supplementary information fig S2

Supplementary information fig S3

Supplementary information fig S4

Supplementary information fig S5

Supplementary information fig S6

Supplementary information fig S7

Supplementary information fig S8

Supplementary information table S1

Supplementary information table S2

Supplementary information table S3

Supplementary information table S4

Supplementary information table S5

## Data Availability

The atomic coordinates and the electron microscopy maps have been deposited in the Protein Data Bank (PDB) under accession number 7D68 and Electron Microscopy Data Bank (EMDB) under accession number EMD-30590, respectively. All relevant data are available from the authors upon request and/or included in the manuscript or Supplementary information.

## References

[1] Drucker DJ, Yusta B (2014). Physiology and pharmacology of the enteroendocrine hormone glucagon-like peptide-2. Annu. Rev. Physiol..

[2] Drucker DJ, Erlich P, Asa SL, Brubaker PL (1996). Induction of intestinal epithelial proliferation by glucagon-like peptide 2. Proc. Natl. Acad. Sci. USA.

[3] Drucker DJ (1997). Regulation of the biological activity of glucagon-like peptide 2 in vivo by dipeptidyl peptidase IV. Nat. Biotechnol..

[4] Austin K, Markovic MA, Brubaker PL (2016). Current and potential therapeutic targets of glucagon-like peptide-2. Curr. Opin. Pharmacol..

[5] Baldassano S, Amato A, Mule F (2016). Influence of glucagon-like peptide 2 on energy homeostasis. Peptides.

[6] Amato A, Baldassano S, Mule F (2016). GLP2: an underestimated signal for improving glycaemic control and insulin sensitivity. J. Endocrinol..

[7] Sasaki-Hamada S, Ito K, Oka J (2013). Neuronal Fos-like immunoreactivity associated with dexamethasone-induced hypertension in rats and effects of glucagon-like peptide-2. Life Sci..

[8] Sasaki-Hamada S, Narusawa K, Nakamura R, Ishibashi H, Oka JI (2018). Effects of centrally administered glucagon-like peptide-2 on blood pressure and barosensitive neurons in spontaneously hypertensive rats. Neuropeptides.

[9] Sasaki-Hamada S, Okada S, Ito K, Iwai T, Oka JI (2012). Immunohistochemical determination of the site of hypotensive effects of glucagon-like peptide-2 in the rat brain. Neuroscience.

[10] Sasaki-Hamada S, Nakamura Y, Koizumi K, Nabeta R, Oka JI (2019). Pharmacological evidence for the relationship between the NMDA receptor and nitric oxide pathway and the antidepressant-like effects of glucagon-like peptide-2 in the mouse forced-swim test. Behav. Brain Res..

[11] Sasaki-Hamada S (2017). Antidepressant-like effects exerted by the intranasal administration of a glucagon-like peptide-2 derivative containing cell-penetrating peptides and a penetration-accelerating sequence in mice. Peptides.

[12] Sasaki-Hamada S, Yuri Y, Hoshi M, Oka JI (2015). Immunohistochemical determination of the site of antidepressant-like effects of glucagon-like peptide-2 in ACTH-treated mice. Neuroscience.

[13] Zhang Y (2017). Cryo-EM structure of the activated GLP-1 receptor in complex with a G protein. Nature.

[14] Liang YL (2017). Phase-plate cryo-EM structure of a class B GPCR-G-protein complex. Nature.

[15] Liang YL (2018). Cryo-EM structure of the active, G_s_-protein complexed, human CGRP receptor. Nature.

[16] Ma S (2020). Molecular basis for hormone recognition and activation of corticotropin-releasing factor receptors.. Mol. Cell.

[17] Liang YL (2018). Phase-plate cryo-EM structure of a biased agonist-bound human GLP-1 receptor-G_s_ complex. Nature.

[18] Qiao A (2020). Structural basis of G_s_ and G_i_ recognition by the human glucagon receptor. Science.

[19] Zhao LH (2019). Structure and dynamics of the active human parathyroid hormone receptor-1. Science.

[20] Liang YL (2020). Toward a structural understanding of class B GPCR peptide binding and activation. Mol. Cell.

[21] Hoare SR (2005). Mechanisms of peptide and nonpeptide ligand binding to Class B G-protein-coupled receptors. Drug Discov. Today.

[22] Duan J (2020). Cryo-EM structure of an activated VIP1 receptor-G protein complex revealed by a NanoBiT tethering strategy. Nat. Commun..

[23] Dixon AS (2016). NanoLuc complementation reporter optimized for accurate measurement of protein interactions in cells. ACS Chem. Biol..

[24] Inoue A (2019). Illuminating G-protein-coupling selectivity of GPCRs. Cell.

[25] Rasmussen SG (2011). Crystal structure of the beta2 adrenergic receptor-G_s_ protein complex. Nature.

[26] Zhou FL (2020). Structural basis for activation of the growth hormone-releasing hormone receptor. Nat. Commun..

[27] Wootten D, Simms J, Miller LJ, Christopoulos A, Sexton PM (2013). Polar transmembrane interactions drive formation of ligand-specific and signal pathway-biased family B G protein-coupled receptor conformations. Proc. Natl. Acad. Sci. USA.

[28] Thulesen J (2002). The truncated metabolite GLP-2(3-33) interacts with the GLP-2 receptor as a partial agonist. Regul. Pept..

[29] Munroe DG (1999). Prototypic G protein-coupled receptor for the intestinotrophic factor glucagon-like peptide 2. Proc. Natl. Acad. Sci. USA.

[30] Liu X (2019). Structural insights into the process of GPCR-G Protein complex formation. Cell.

[31] Graaf C (2016). Glucagon-like peptide-1 and its class B G protein-coupled receptors: a long march to therapeutic successes. Pharmacol. Rev..

[32] Ma H., et al. Structural insights into the activation of GLP-1R by a small molecule agonist. *Cell Res*. 10.1038/s41422-020-0384-8 (2020). 10.1038/s41422-020-0384-8 PMC7784854 32724086

[33] Wu F (2020). Full-length human GLP-1 receptor structure without orthosteric ligands. Nat. Commun..

[34] Jazayeri A (2017). Crystal structure of the GLP-1 receptor bound to a peptide agonist. Nature.

[35] Song G (2017). Human GLP-1 receptor transmembrane domain structure in complex with allosteric modulators. Nature.

[36] Zhang H (2017). Structure of the full-length glucagon class B G-protein-coupled receptor. Nature.

[37] Ehrenmann J (2018). High-resolution crystal structure of parathyroid hormone 1 receptor in complex with a peptide agonist. Nat. Struct. Mol. Biol..

[38] Zhang H (2018). Structure of the glucagon receptor in complex with a glucagon analogue. Nature.

[39] Zhao LH (2016). Differential requirement of the extracellular domain in activation of class B G protein-coupled receptors. J. Biol. Chem..

[40] Lee C (1994). Role of the extracellular regions of the parathyroid hormone (PTH)/PTH-related peptide receptor in hormone binding. Endocrinology.

[41] Kobayashi K (2020). Cryo-EM structure of the human PAC1 receptor coupled to an engineered heterotrimeric G protein. Nat. Struct. Mol. Biol..

[42] Wang J (2020). Cryo-EM structures of PAC1 receptor reveal ligand binding mechanism. Cell Res..

[43] Liu P (2016). The structural basis of the dominant negative phenotype of the Galphai1beta1gamma2 G203A/A326S heterotrimer. Acta Pharmacol. Sin..

[44] Zheng SQ (2017). MotionCor2: anisotropic correction of beam-induced motion for improved cryo-electron microscopy. Nat. Methods..

[45] Zhang K (2016). Gctf: Real-time CTF determination and correction. J. Struct. Biol..

[46] Scheres SH (2012). RELION: implementation of a Bayesian approach to cryo-EM structure determination. J. Struct. Biol..

[47] Heymann JB (2018). Guidelines for using Bsoft for high resolution reconstruction and validation of biomolecular structures from electron micrographs. Protein Sci..

[48] Waterhouse A (2018). SWISS-MODEL: homology modelling of protein structures and complexes. Nucleic Acids Res..

[49] Pettersen EF (2004). UCSF Chimera–a visualization system for exploratory research and analysis. J. Comput. Chem..

[50] Emsley P, Cowtan K (2004). Coot: model-building tools for molecular graphics. Acta Crystallogr. D Biol. Crystallogr..

[51] Adams PD (2010). PHENIX: a comprehensive Python-based system for macromolecular structure solution. Acta Crystallogr. D Biol. Crystallogr..

[52] Jensen PC, Thiele S, Ulven T, Schwartz TW, Rosenkilde MM (2008). Positive versus negative modulation of different endogenous chemokines for CC-chemokine receptor 1 by small molecule agonists through allosteric versus orthosteric binding. J. Biol. Chem..

[53] Mitternacht S (2016). FreeSASA: an open source C library for solvent accessible surface area calculations. F1000Res..

[54] Lomize MA, Lomize AL, Pogozheva ID, Mosberg HI (2006). OPM: orientations of proteins in membranes database. Bioinformatics.

