# Supplementary material for: A unique hormonal recognition feature of the human glucagon-like peptide-2 receptor
Source: Cell Res. 2020 Nov 25;30(12):1098–108. doi: 10.1038/s41422-020-00442-0 (PMC7785020; doi:10.1038/s41422-020-00442-0)
Supplement: Supplementary file 1 — Supplementary information fig S1 [file 41422_2020_442_MOESM1_ESM.pdf]

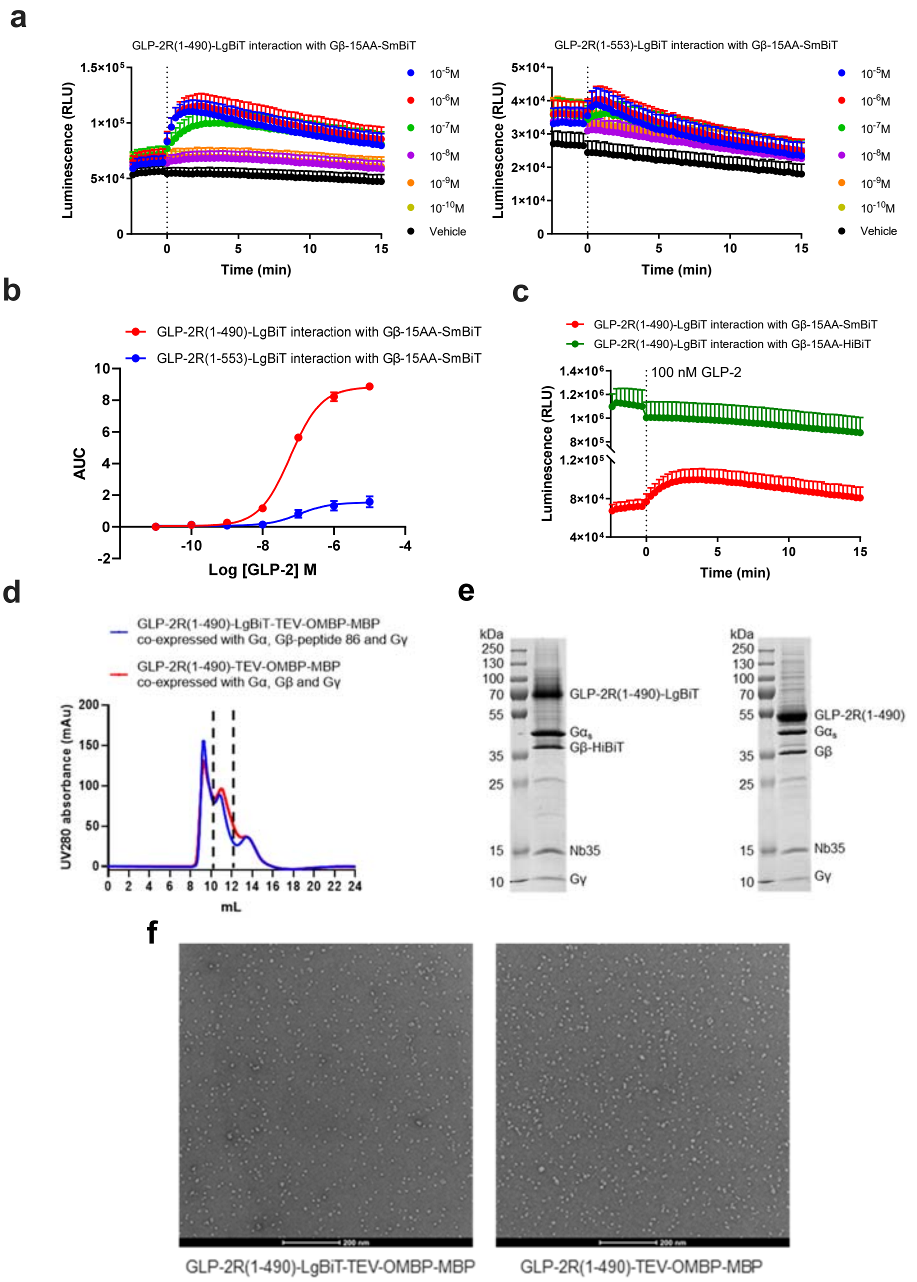

Supplementary information, Fig. S1 | NanoBiT strategy used in protein purification. **a**, Wild-type GLP-2R(1-553)-LgBiT and truncated GLP-2R(1-490)-LgBiT were transiently transfected to HEK 293T cells, respectively, and their responses to GLP-2 (0.1 nM to 10 μM) detected by luminescence. The dashed line indicates the time point of GLP-2 addition. **b**, Concentration-response curves are expressed as AUC (0–15 min) for each concentration. **c**, SmBiT was replaced by HiBiT to examine the difference in GLP-2R interaction with G protein. The dashed line indicates the time point of 100 nM GLP-2 addition. Data are displayed as means ± S.E.M. of at least three independent experiments performed in duplicate. **d**, Comparison of size-exclusion chromatography results with (blue line) and without (red line) NanoBiT strategy on Superdex 200 Increase 10/30 column. The peaks at 9 ml, 11 ml and 13 ml show the complex aggregation, complex monomer and partial dissociation, respectively. **e**, SEC fractions containing hGLP-2R–G_s monomeric complex (shown as dash lines in **d**) were collected and analyzed by SDS-PAGE with (left) and without (right) NanoBiT strategy using Coomassie blue staining. **f**, Representative negative staining profiles with (left) and without NanoBiT strategy (right).
