# Supplementary material for: A unique hormonal recognition feature of the human glucagon-like peptide-2 receptor
Source: Cell Res. 2020 Nov 25;30(12):1098–108. doi: 10.1038/s41422-020-00442-0 (PMC7785020; doi:10.1038/s41422-020-00442-0)
Supplement: Supplementary file 2 — Supplementary information fig S2 [file 41422_2020_442_MOESM2_ESM.pdf]

a

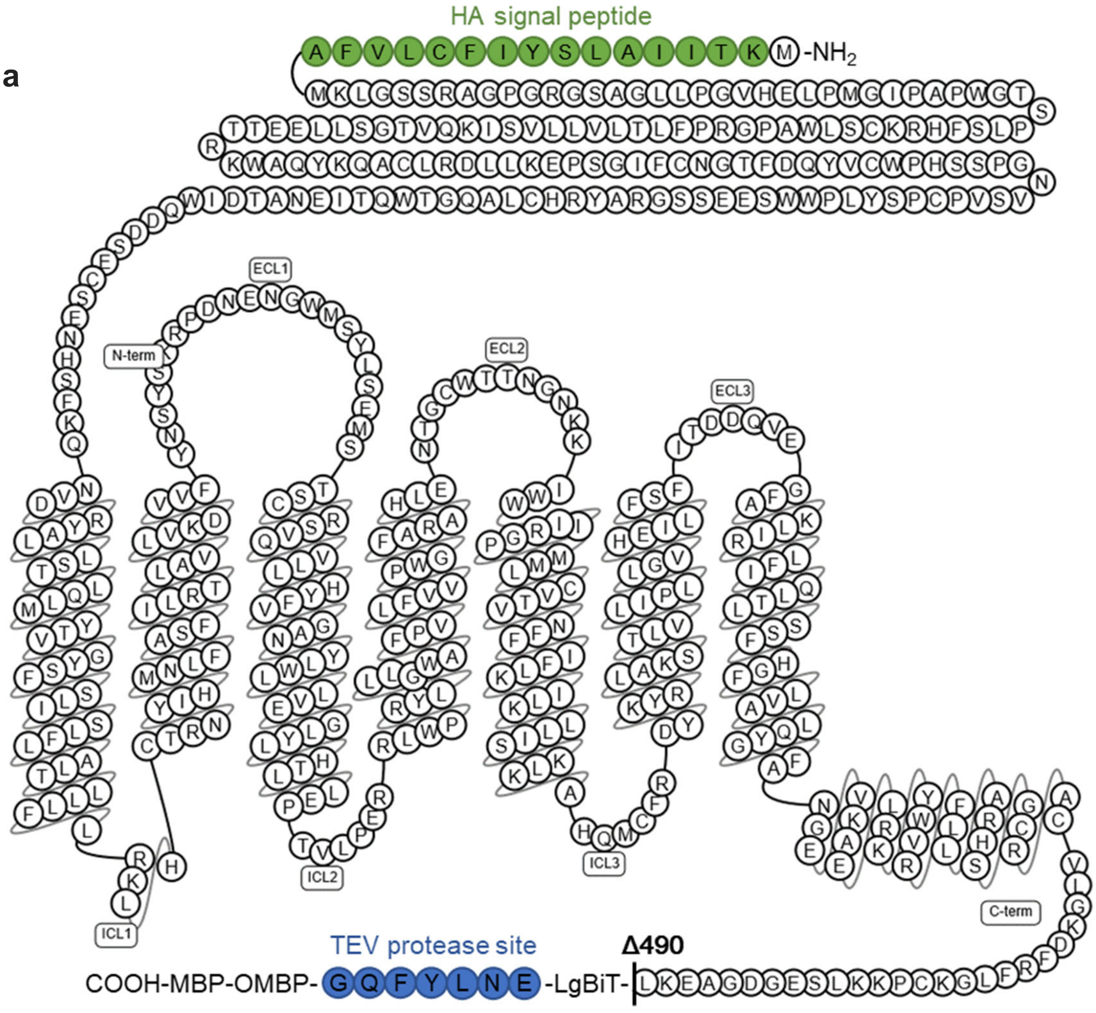

b

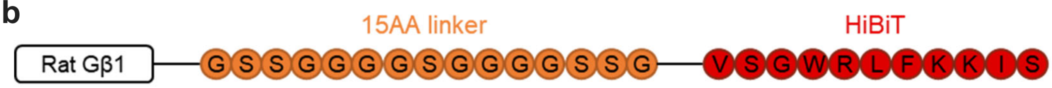

Supplementary information, Fig. S2 | GLP-2R and Gβ constructs used for structure determination. a, Since the human GLP-2R has no predicted signal peptide, a HA signal peptide (green) was directly added to the receptor N-terminus. GLP-2R was truncated to the 490th amino acid and attached to LgBiT without linker. The C-terminus was modified with a TEV protease site (blue) and an OMBP-MBP tag. **b**, Rat Gβ1 was attached to HiBiT (red) with a 15-amino acid (15AA) linker (orange) between them. N-term, N-terminal; C-term, C-terminal; 15AA, 15-amino acid.
