# Supplementary material for: A unique hormonal recognition feature of the human glucagon-like peptide-2 receptor
Source: Cell Res. 2020 Nov 25;30(12):1098–108. doi: 10.1038/s41422-020-00442-0 (PMC7785020; doi:10.1038/s41422-020-00442-0)
Supplement: Supplementary file 3 — Supplementary information fig S3 [file 41422_2020_442_MOESM3_ESM.pdf]

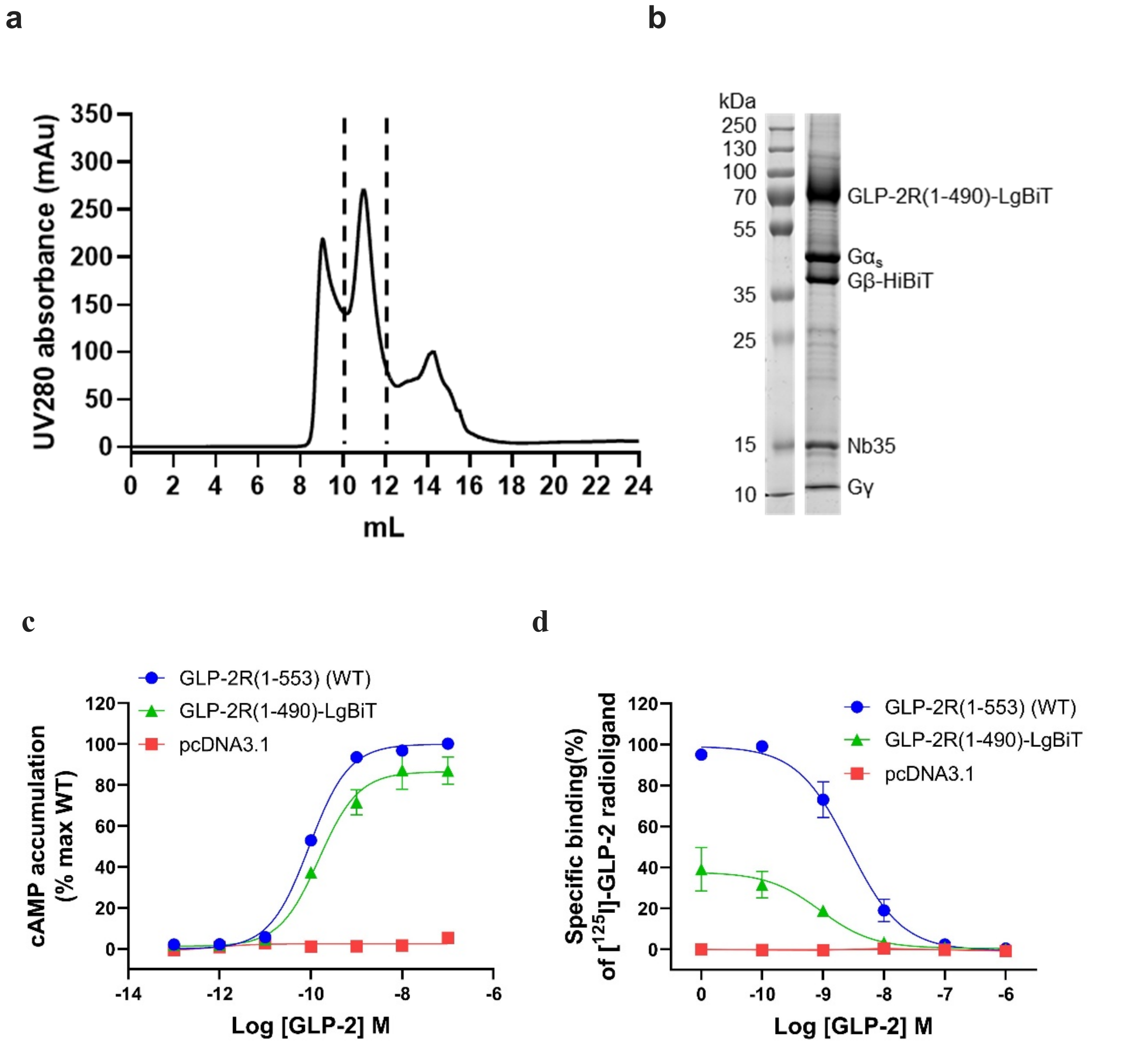

Supplementary information, Fig. S3 | Purification and bioactivity of the GLP-2–GLP-2R–G_s complex. a, Final size-exclusion chromatography result of the complex on Superdex 200 Increase 10/30 column. The peaks at 9 ml, 11 ml and 13 ml how the complex aggregation, complex monomer and partial dissociation, respectively. **b,** SEC fractions containing hGLP-2R–G_s monomeric complex (shown as dash lines in **a**) are collected and analyzed by SDS-PAGE using Coomassie blue staining. **c**, cAMP accumulation in wild-type (WT) and truncated GLP-2R expressing cells following cognate ligand stimulation. **d**, Radiolabeled ligand binding properties of WT and truncated GLP-2 receptors. max, maximum response.
