# Supplementary material for: A unique hormonal recognition feature of the human glucagon-like peptide-2 receptor
Source: Cell Res. 2020 Nov 25;30(12):1098–108. doi: 10.1038/s41422-020-00442-0 (PMC7785020; doi:10.1038/s41422-020-00442-0)
Supplement: Supplementary file 4 — Supplementary information fig S4 [file 41422_2020_442_MOESM4_ESM.pdf]

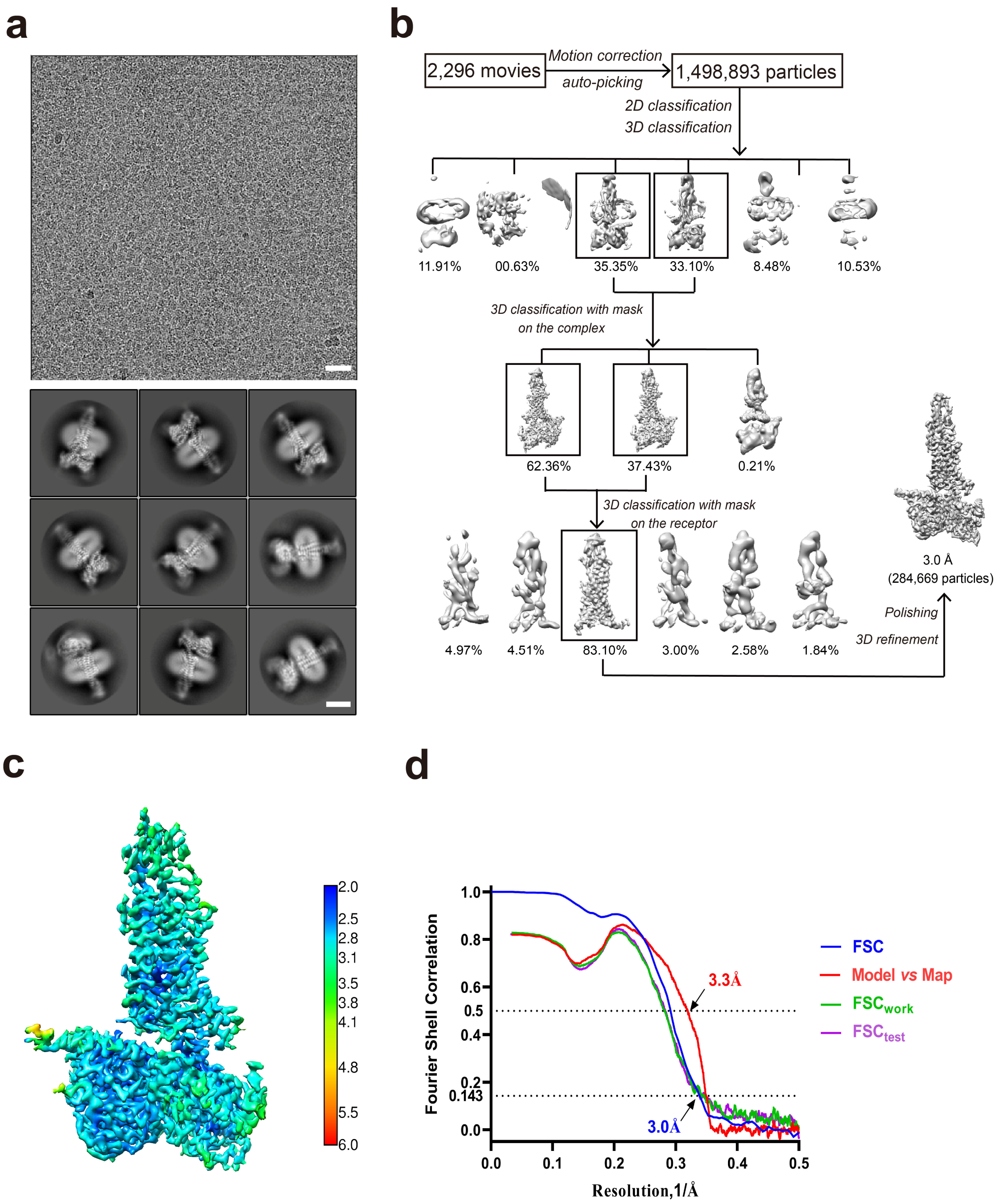

Supplementary information, Fig. S4 | Cryo-EM analysis of the GLP-2–GLP-2R–G_s complex. **a**, Cryo-EM micrograph (scale bar: 40 nm) and representative two-dimensional class averages of the active GLP-2–GLP-2R–G_s complex in LMNG detergent micelles (scale bar: 5 nm). **b**, Flow chart of cryo-EM data processing. Details are described in Method section. **c**, Local resolution distribution map of the GLP-2–GLP-2R–G_s structure. **d**, Gold-standard Fourier shell correlation (FSC) curves of overall refined receptor. Final three-dimensional cryo-EM map calculated in RELION after auto-refinement.
