# Supplementary material for: A unique hormonal recognition feature of the human glucagon-like peptide-2 receptor
Source: Cell Res. 2020 Nov 25;30(12):1098–108. doi: 10.1038/s41422-020-00442-0 (PMC7785020; doi:10.1038/s41422-020-00442-0)
Supplement: Supplementary file 5 — Supplementary information fig S5 [file 41422_2020_442_MOESM5_ESM.pdf]

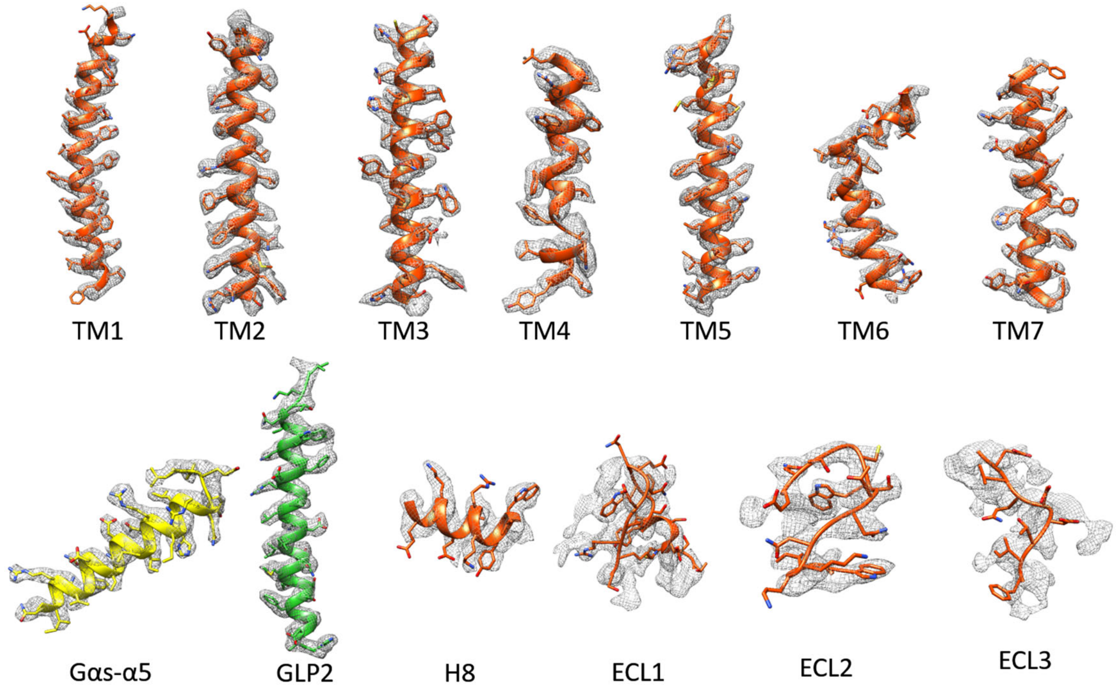

Supplementary information, Fig. S5 | Atomic-resolution model of the GLP-2–GLP-2R–G_s complex in the cryo-EM density map. EM density map and model are shown for all seven transmembrane α-helices, helix 8 and all extracellular loops of GLP-2R, GLP-2 and the α5-helix of the $G\alpha_s$ Ras-like domain.
