# Supplementary material for: A unique hormonal recognition feature of the human glucagon-like peptide-2 receptor
Source: Cell Res. 2020 Nov 25;30(12):1098–108. doi: 10.1038/s41422-020-00442-0 (PMC7785020; doi:10.1038/s41422-020-00442-0)
Supplement: Supplementary file 6 — Supplementary information fig S6 [file 41422_2020_442_MOESM6_ESM.pdf]

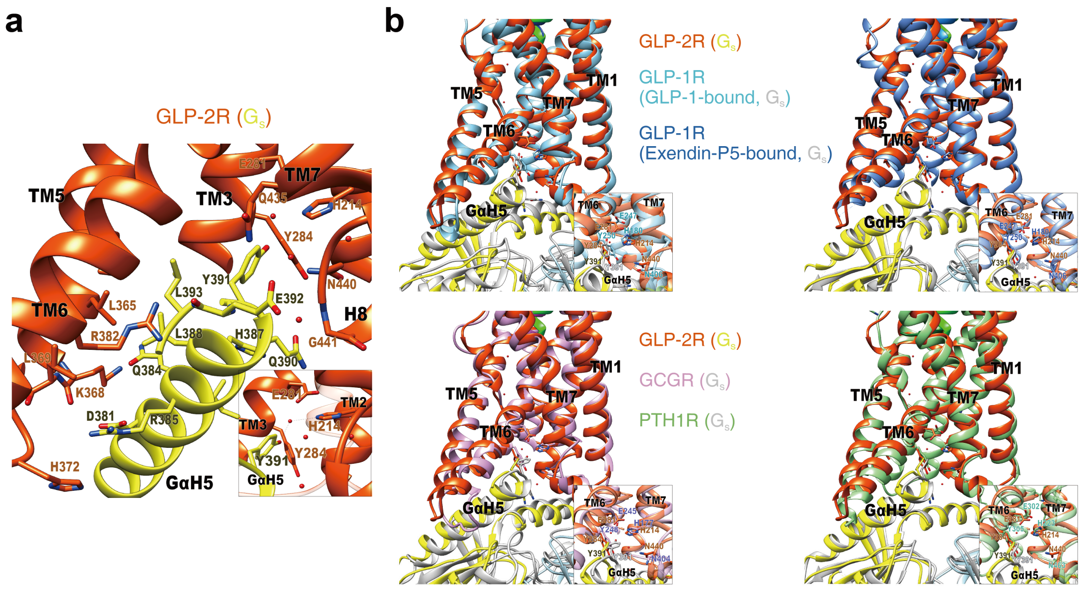

Supplementary information, Fig. S6 | Comparison of G protein coupling by GLP-2R and other class B GPCRs. a, G protein coupling by GLP-2R. The α5-helix (GαH5) of the Gαs Ras-like domain inserts into an intracellular crevice of GLP-2R TMD. Water molecules are shown as spheres in red and hydrogen bonds involved are shown as dashed lines. **b,** Superposition of GLP-2–GLP-2R–Gs with other class B receptor–G protein complexes that aligned based on the TMs 2, 3 and 4. The interaction residues are shown as sticks. Close views depict the receptor-GαH5 interface.
