# Supplementary material for: A unique hormonal recognition feature of the human glucagon-like peptide-2 receptor
Source: Cell Res. 2020 Nov 25;30(12):1098–108. doi: 10.1038/s41422-020-00442-0 (PMC7785020; doi:10.1038/s41422-020-00442-0)
Supplement: Supplementary file 7 — Supplementary information fig S7 [file 41422_2020_442_MOESM7_ESM.pdf]

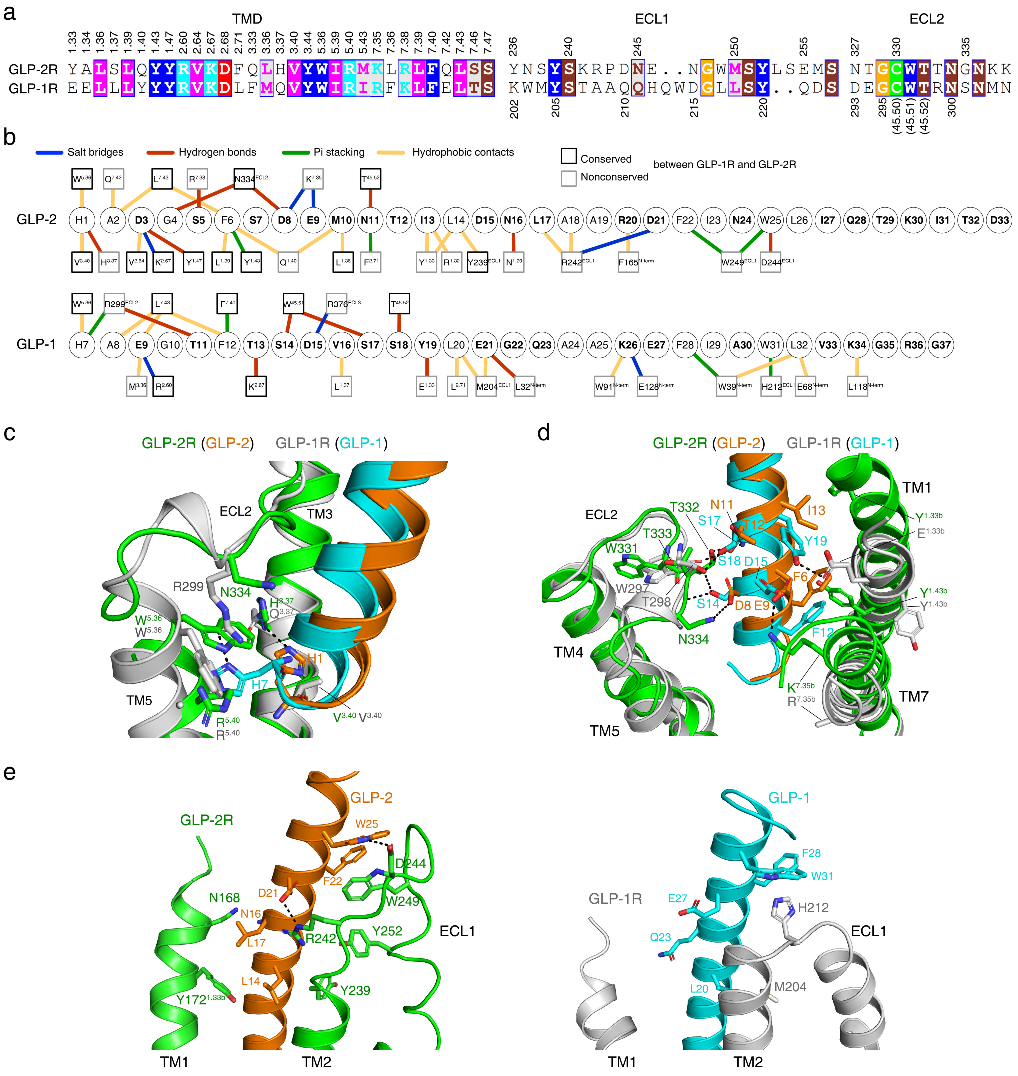

Supplementary information, Fig. S7 | Comparison of peptide-binding modes between GLP-2R and GLP-1R. a, Sequence alignment of the binding pocket residues of GLP-2R and GLP-1R. Receptor residues are numbered according to Wootten numbering system[27]. For residues constituting the peptide-binding pocket, except ECL1, the transmembrane helices and ECL2 have a sequence identity of 57.6% and 58.3%, respectively. **b**, Comparison of the peptide-receptor interaction diagrams between GLP-2R and GLP-1R. Conserved residues between GLP-2R and GLP-1R are shown in black box, and divergent residues are shown in gray box. Bold residues in peptide indicate they are distinct between GLP-2 and GLP-1. **c-e**, Pairwise comparison of the peptide binding modes of GLP-2R and GLP-1R with hydrogen bonds shown as dotted lines. Close-up views of the interactions between the receptor and the N-terminus (**c**), middle region (**d**) and C-terminus (**e**) of the peptide.
