# Supplementary material for: A unique hormonal recognition feature of the human glucagon-like peptide-2 receptor
Source: Cell Res. 2020 Nov 25;30(12):1098–108. doi: 10.1038/s41422-020-00442-0 (PMC7785020; doi:10.1038/s41422-020-00442-0)
Supplement: Supplementary file 8 — Supplementary information fig S8 [file 41422_2020_442_MOESM8_ESM.pdf]

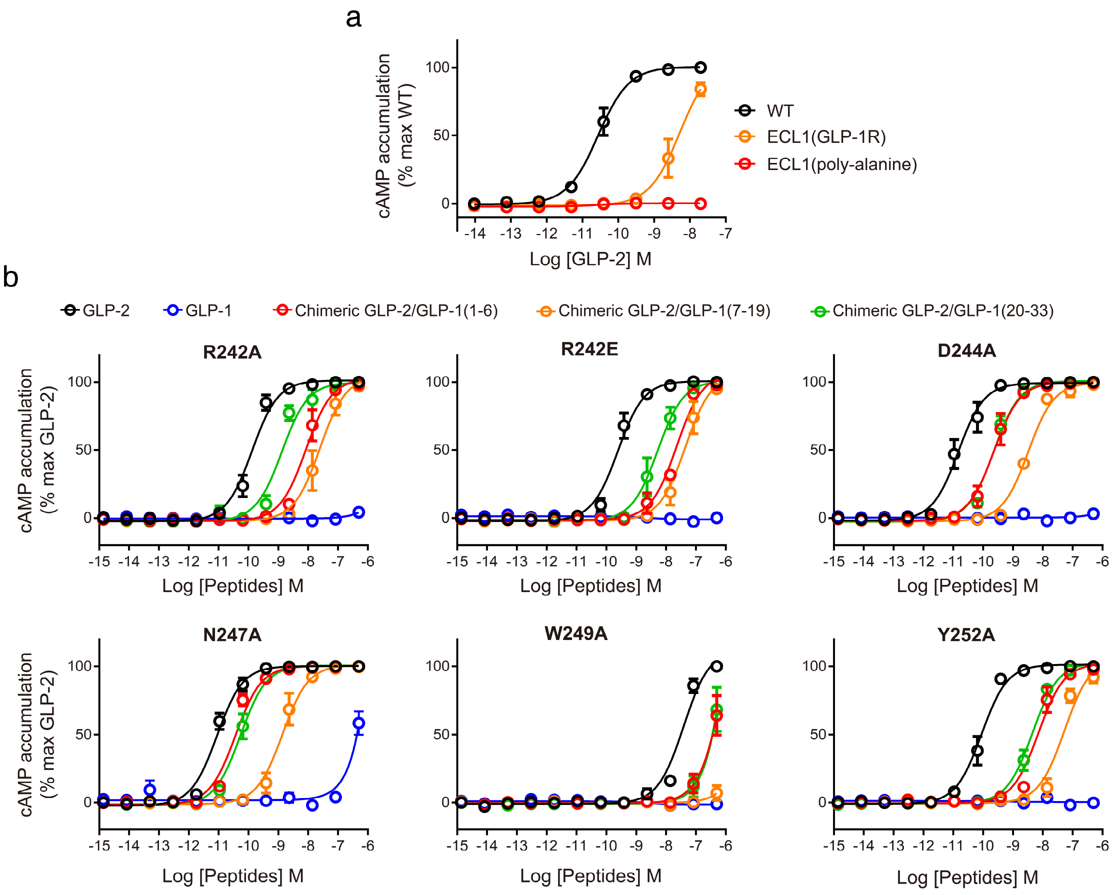

Supplementary information, Fig. S8 | Signaling profiles of chimeric GLP-2/GLP-1 peptides on the ECL1 mutants. a. cAMP accumulation in WT and ECL1-substituted GLP-2R expressing cells. ECL1 of GLP-2R mutants (residues 236-257) substituted with the corresponding segment of GLP-1R or poly-alanine are labeled as ECL1(GLP-1R) and ECL1(poly-alanine), respectively. **b**, cAMP accumulation in WT and ECL1 mutated (single-point) GLP-2R expressing cells. Replacements of the N-terminus (residues 1-6), the middle region (residues 7-19) and the C-terminus (residues 20-33) of GLP-2 by corresponding segments of GLP-1 are labeled as chimeric GLP-2/GLP-1(1-6), GLP-2/GLP-1(7-19) and GLP-2/GLP-1(20-33), respectively. Dose-response curves were generated and graphed as means ± S.E.M. from three independent experiments.
