# Supplementary material for: A unique hormonal recognition feature of the human glucagon-like peptide-2 receptor
Source: Cell Res. 2020 Nov 25;30(12):1098–108. doi: 10.1038/s41422-020-00442-0 (PMC7785020; doi:10.1038/s41422-020-00442-0)
Supplement: Supplementary file 9 — Supplementary information table S1 [file 41422_2020_442_MOESM9_ESM.pdf]

Supplementary information, Table. S1 | Cryo-EM data collection, refinement and validation statistics.

GLP-2-GLP-2R-G_s complex	Value
**Data collection and processing**	
Magnification	49310
Voltage (kV)	300
Electron exposure ($e^-/Å^2$)	64
Defocus range (μm)	-0.5 ~ -2.5
Pixel size (Å)	1.014
Symmetry imposed	C1
Initial particle projections (no.)	1,498,893
Final particle projections (no.)	284,669
Map resolution (Å)	3.0
FSC threshold	0.143
Map resolution range (Å)	2.5-5.0
**Refinement**	
Initial model used (PDB code)	6NBF
Model resolution (Å)	3.1
FSC threshold	0.5
Model resolution range (Å)	2.5-5.0
Map sharpening *B* factor ($Å^2$)	-94.5881
Model composition	
Non-hydrogen atoms	8566
Protein residues	1070
Water	7
B factors ($Å^2$)	
Protein	58.72
Water	30.00
R.M.S. deviations	
Bond lengths (Å)	0.004
Bond angles (°)	0.648
Validation	
MolProbity score	1.77
Clash score	7.98
Poor rotamers (%)	0.76
Ramachandran plot	
Favored (%)	95.15
Allowed (%)	4.75
Disallowed (%)	0.10
