# Supplementary material for: A unique hormonal recognition feature of the human glucagon-like peptide-2 receptor
Source: Cell Res. 2020 Nov 25;30(12):1098–108. doi: 10.1038/s41422-020-00442-0 (PMC7785020; doi:10.1038/s41422-020-00442-0)
Supplement: Supplementary file 10 — Supplementary information table S2 [file 41422_2020_442_MOESM10_ESM.pdf]

Supplementary information, Table. S2 | Effects of GLP-2-mediated cAMP accumulation and binding affinity[a].

**Receptor**	**cAMP accumulation**		**Binding**
	**pEC_{50} ± SEM**	**E_{max} (% WT[b])**	**pIC_{50} ± SEM**
GLP-2R (1-553) (WT)	10.0 ± 0.04	100	8.5 ± 0.07
GLP-2R (1-490)-LgBiT	9.8 ± 0.1	85.3 ± 4.0*	9.1 ± 0.3

[a]All data were fitted with a three-parameter logistic curve to obtain pEC_{50} and pIC_{50} values. Data represent means ± S.E.M. of at least three independent experiments performed duplicate. Statistical significance was determined with a two-tailed Student's *t*-test. *$P < 0.05$.

[b] WT, wild-type.
