# Supplementary material for: A unique hormonal recognition feature of the human glucagon-like peptide-2 receptor
Source: Cell Res. 2020 Nov 25;30(12):1098–108. doi: 10.1038/s41422-020-00442-0 (PMC7785020; doi:10.1038/s41422-020-00442-0)
Supplement: Supplementary file 11 — Supplementary information table S3 [file 41422_2020_442_MOESM11_ESM.pdf]

Supplementary information, Table. S3 | Effects of residue mutation in the ligand-binding pocket on GLP-2R-induced cAMP accumulation[a].

	Human GLP-2(1-33)	
**Receptor mutant**	**pEC_{50}±SEM**	**E_{max} (% WT[b])**
GLP-2R (1-553) (WT)	10.4±0.05	100.9±2.4
$Y182^{1.43}A$	9.6±0.08	101.8±3.9
$Y186^{1.47}A$	9.2±0.06**	100.3±2.8
$K231^{2.67}A$	9.9±0.07	105.1±3.0
$R242^{ECL1}E$	9.1±0.03**	108.2±1.9
$R242^{ECL1}A$	9.8±0.07	106.7±3.4
$W249^{ECL1}A$	7.2±0.7***	127.9±161.5
$Y252^{ECL1}A$	9.8±0.06	103.1±2.6
$H268^{3.37}A$	7.8±0.2***	118.4±22.6
$N334^{ECL2}A$	9.6±0.06	102.7±2.9
$W340^{5.36}A$	8.9±0.06***	106.8±3.6
$R344^{5.40}A$	9.2±0.05**	108.6±2.8
$K414^{7.35}A$	9.8±0.07	110.0±3.3

[a]All data were fitted with a three-parameter logistic curve to obtain pEC_{50} values. Data represent means ± S.E.M. of at least three independent experiments performed duplicate. One-way ANOVA and Dunnett's post-test were used to determine statistical difference. *P<0.05, **P<0.01, ***P<0.001.

[b] WT, wild-type.
