# Supplementary material for: A unique hormonal recognition feature of the human glucagon-like peptide-2 receptor
Source: Cell Res. 2020 Nov 25;30(12):1098–108. doi: 10.1038/s41422-020-00442-0 (PMC7785020; doi:10.1038/s41422-020-00442-0)
Supplement: Supplementary file 12 — Supplementary information table S4 [file 41422_2020_442_MOESM12_ESM.pdf]

Supplementary information, Table. S4 | Effects of residue mutation in the ECL1 on GLP-2R-induced cAMP accumulation[a].

	Human GLP-2(1-33)	
**Receptor mutant**	**pEC_{50}±SEM**	**E_{max} (% WT[b])**
GLP-2R (1-553) (WT)	10.6±0.07	101.0±2.9
ECL1(GLP-1R) [c]	10.6±0.9	2.5±0.9***
ECL1(poly-alanine) [c]	8.3±0.1*	108.4±10.6

[a]All data were fitted with a three-parameter logistic curve to obtain pEC_{50} values. Data represent means ± S.E.M. of at least three independent experiments performed duplicate. One-way ANOVA and Dunnett's post-test were used to determine statistical difference. *P<0.05, **P<0.01, ***P<0.001.

[b] WT, wild-type.

[c] ECL1 of GLP-2R mutants (residues 236-257) substituted with the corresponding segment of GLP-1R or poly-alanine are labeled as ECL1(GLP-1R) and ECL1(poly-alanine), respectively.
