# Supplementary material for: A unique hormonal recognition feature of the human glucagon-like peptide-2 receptor
Source: Cell Res. 2020 Nov 25;30(12):1098–108. doi: 10.1038/s41422-020-00442-0 (PMC7785020; doi:10.1038/s41422-020-00442-0)
Supplement: Supplementary file 13 — Supplementary information table S5 [file 41422_2020_442_MOESM13_ESM.pdf]

Supplementary information, Table. S5 | Effects of residue mutation in ECL1 on GLP-2R-induced cAMP accumulation by different ligands[a].

	GLP-2(1-33)		GLP-1(1-31)		Chimeric GLP-1/GLP-2 (1-6)		Chimeric GLP-1/GLP-2 (7-19)		Chimeric GLP-1/GLP-2 (20-33)	
**Receptor mutant**	**pEC_{50} ±SEM**	**E_{max} (% WT[b])**	**pEC_{50} ±SEM**	**E_{max} (% WT)**	**pEC_{50} ±SEM**	**E_{max} (% WT)**	**pEC_{50} ±SEM**	**E_{max} (% WT)**	**pEC_{50} ±SEM**	**E_{max} (% WT)**
GLP-2R (WT)	10.8±0.04	103.0±1.6	N.D. [c]	N.D.	9.8±0.05***	102.9±1.8	8.5±0.06***	103.2±2.6	9.9±0.04***	103.8±1.5
GLP-1R (WT)	8.3±1.1*	4.0±3.1***	10.9±0.06	106.2±2.7	8.1±0.6*	7.1±3.2***	10.9±0.06	104.7±2.4	8.5±0.04*	112.1±2.8
GLP-2R R242^{ECL1}E	9.6±0.06	103.0±2.3	8.6±0.9	-2.2±0.9***	7.6±0.06*	108.3±3.2	7.4±0.09*	107.8±5.5	8.3±0.07	103.2±3.5
GLP-2R R242^{ECL1}A	9.9±0.06	103.6±2.3	N.D.	N.D.	8.1±0.07***	105.0±3.2	7.6±0.09***	106.4±4.9	8.9±0.08***	102.0±3.1
GLP-2R D244^{ECL1}A	10.8±0.07	101.2±2.8	N.D.	N.D.	9.6±0.06***	102.0±2.5	8.5±0.04***	102.0±1.8	9.6±0.05***	103.6±1.8
GLP-2R N247^{ECL1}A	11.1±0.05	102.6±1.8	N.D.	N.D.	10.4±0.05***	101.9±1.8	8.9±0.07***	102.2±2.8	10.3±0.05***	102.9±1.8
GLP-2R W249^{ECL1}A	7.4±0.07	113.2±4.1	8.6±0.7	-2.4±0.7	5.9±0.6	226.4±219.9	N.D.	N.D.	5.2±2.8	953.1±5657.0
GLP-2R Y252^{ECL1}A	10.1±0.05	102.3±2.2	9.2±3.6	-0.7±1.1***	8.2±0.07	102.8±3.1	7.3±0.08	106.4±5.1	8.4±0.05	103.7±2.1

[a]All data were fitted with a three-parameter logistic curve to obtain pEC_{50} values. Data represent means ± S.E.M. of at least three independent experiments performed duplicate. One-way ANOVA and Dunnett's post-test were used to determine statistical difference. *P<0.05, **P<0.01, ***P<0.001.

[b] WT, wild-type.
